# Cloning of carrier cells infected with oncolytic adenovirus driven by *midkine* promoter and biosafety studies

**DOI:** 10.1002/jgm.3064

**Published:** 2019-02-01

**Authors:** Katsuyuki Hamada, Soichi Takagi, Hajime Kuboshima, Hideaki Shimada, Kazuko Takagi, Toshiaki Yasuoka, Keiichi Matsubara, Yukiko Sassa, Tetsuya Furuya, Kazuhiko Suzuki, Tsuyoshi Uchide, Tetsuya Mizutani, Kenzaburo Tani, Hiroshi Itoh, Takashi Sugiyama

**Affiliations:** ^1^ Department of Clinical Oncology, School of Medicine Toho University Tokyo Japan; ^2^ Department of Obstetrics and Gynecology, School of Medicine Ehime University Ehime Japan; ^3^ Advanced Biomedical Engineering and Science, Graduate School of Medicine Tokyo Women's Medical University Tokyo Japan; ^4^ Animal Stem Cell Inc. Tokyo Japan; ^5^ Department of Surgery, School of Medicine Toho University Tokyo Japan; ^6^ Cooperative Department of Veterinary Medicine, Faculty of Agriculture Tokyo University of Agriculture and Technology Tokyo Japan; ^7^ Research and Education Center for Prevention of Global Infectious Disease of Animal, Faculty of Agriculture Tokyo University of Agriculture and Technology Tokyo Japan; ^8^ Project Division of ALA Advanced Medical Research, The Institute of Medical Science The University of Tokyo Tokyo Japan; ^9^ Animal Medical Center, Faculty of Agriculture Tokyo University of Agriculture and Technology Tokyo Japan

**Keywords:** adenovirus (AdV), cell therapy, gene delivery, gene therapy, oncology, ovarian cancer, tumor therapy

## Abstract

**Background:**

A549 carrier cells infected with oncolytic adenovirus can induce complete tumor reduction of subcutaneous ovarian tumors but not intraperitoneal disseminated ovarian tumors. This appears to be a result of the insufficient antitumor effect of A549 carrier cells. Therefore, in the present study, we cloned a novel carrier cell with the aim of improving the antitumor effects.

**Methods:**

Carrier cells infected with oncolytic adenovirus AdE3‐*midkine* with a *midkine* promoter were cloned by limiting dilution. We examined the antitumor effects of these cells on subcutaneous and intraperitoneal OVHM ovarian tumors in a syngeneic mouse model. Biosafety tests were conducted in beagle dogs and rabbits.

**Results:**

We cloned EHMK‐51‐35 carrier cells with 10‐fold higher antitumor effects compared to A549 carrier cells *in vitro*. EHMK‐51‐35 carrier cells co‐infected with AdE3‐*midkine* and Ad‐*mGM‐CSF* induced a 100% complete tumor reduction in subcutaneous tumors and a 60% reduction of intraperitoneal disseminated tumors. Single‐dose acute toxicity test on beagle dogs with EHMK‐51‐35 carrier cells co‐infected with AdE3‐*midkine* and Ad‐*cGM‐CSF* showed no serious side effects. Biologically active adenoviruses were not detected in the blood, saliva, feces, urine or whole organs. In a chronic toxicity test, VX2 tumors in rabbits were injected five times with EHMK‐51‐35 carrier cells infected with AdE3‐*midkine* and these rabbits showed no serious side effects.

**Conclusions:**

Significant antitumor effects and safety of cloned EHMK‐51‐35 carrier cells were confirmed in intraperitoneal ovarian tumors and toxicity tests, respectively. These findings will be extended to preclinical efficacy studies using dogs and cats, with the aim of conducting human clinical trials on refractory solid tumors.

## 1 INTRODUCTION

More than 1000 clinical trials of cancer gene therapies have been conducted to date, although encouraging clinical results have yet to be obtained.^1^ Replication‐competent viral vectors have been developed to improve antitumor activity. Talimogene laherparepvec is a genetically modified herpes simplex virus type 1 that expresses granulocyte‐macrophage colony‐stimulating factor (GM‐CSF) and was shown to improve the durable response rate in a phase III single‐agent registration study.^2^ This viral therapy is the first gene therapy drug approved by the US Food and Drug Administration. An oncolytic, nonpathogenic ECHO‐7 virus without genetic modification (Rigvir) was shown to significantly prolong survival in patients with early‐stage melanoma without any side effects.^3^ However, these viral vectors do not show a significant clinical effect with respect to improving the survival rate of patients with advanced cancers because they result in high titers of neutralizing antibodies that inhibit repetitive viral infections.^4^ Adenovirus infection is highly likely to be inhibited even at initial administration because adenovirus type 5 seroprevalence is 80%–90% in humans.^5^ To evade such anti‐adenovirus immunity, adenovirus can be coated with liposomes or polymers. However, the infectivity of these modified adenoviruses is effective only in low antibody titers, and these conditions have little antitumor effect *in vivo* and fail to induce complete tumor reduction.^6^, ^7^ Furthermore, because the adenovirus may induce fatal side effects as a result of a cytokine surge,^8^ it cannot be administered intravenously. However, carrier cells infected with oncolytic adenovirus can be safely administered intravenously with significant antitumor effects.^9^

Many studies of replication‐competent virus‐infected carrier cells have been described, including PA‐1 ovarian cancer cells infected with oncolytic HSV‐1,^10^ mesenchymal stem cells infected with oncolytic adenovirus,^11^ myeloma cells infected with oncolytic measles and vaccinia viruses^12^ and autologous CD8^+^ lymphocytes infected with oncolytic vesicular stomatitis virus.^13^ However, the anti‐tumor potency of these carrier cells has been insufficient because they cannot produce sufficiently high virus titers and are vulnerable to damage even before targeting cancer cells.

Human non‐small cell lung cancer A549 cells have been conventionally used to produce various viruses including adenovirus because of their high virus production capacity. A previous study showed that A549 carrier cells infected with oncolytic adenovirus exhibited a significant antitumor effect in immunocompromised mice.^14^ Adenoviral particle‐containing cell fragments derived from these A549 carrier cells were shown to be engulfed by target cancer cells.^14^ This novel non‐receptor‐mediated adenoviral infection system circumvents neutralization by anti‐adenovirus antibodies and enhances antitumor activity by inducing anti‐adenoviral cytotoxic T lymphocyte (CTL) responses after pre‐immunization with adenovirus in immunocompetent mice, thus inducing an anti‐tumoral immune response. However, although A549 carrier cells infected with oncolytic adenovirus could completely reduce subcutaneous ovarian tumors, they were unable to reduce intraperitoneally disseminated ovarian tumors.

Biosafety tests for ovarian cancer‐specific *IAI.3B* promoter‐driven oncolytic adenovirus‐infected A549 carrier cells for human clinical trial of recurrent solid tumors were reported in mice and rabbits.^15^ However, biosafety tests for carrier cells co‐infected with oncolytic adenovirus and adenovirus‐*GM‐CSF* have yet to be reported.

*Midkine* is overexpressed in the malignant solid tumors of humans, dogs and cats. More than one hundred million dogs and cats are bred in developed countries such as Japan, the USA and Europe, and half of animal deaths are the result of cancers.^16^ Because treating cancers in companion animals by surgery, radiation and chemotherapy is impractical and uneconomical, more convenient and less invasive treatment methods should be developed. Complete treatment of tumors in companion animals by injection of carrier cells might be a potential strategy to circumvent these problems.

In the present study, to induce complete tumor reduction of intraperitoneally disseminated ovarian tumors using carrier cells infected with oncolytic adenovirus, we cloned a new carrier cell from cells that were established in our laboratory and characterized the antitumor activity and biosafety of these carrier cells. We injected the newly developed cloned carrier cells infected with *midkine* promoter‐driven oncolytic adenovirus into mice, beagle dogs and rabbits aiming to examine antitumor efficacy and biosafety. These efficacy and biosafety tests could comprise a preliminary study for a clinical efficacy trial regarding recurrent canine and feline solid tumors and potentially provide proof‐of‐concept for their use as a pre‐clinical efficacy trial for testing in humans.

## 2 MATERIALS AND METHODS

### 2.1 Cell lines and adenoviruses

Human ovarian cancer HEY and non‐small cell lung cancer A549 cells were cultured in RPMI, and murine ovarian carcinoma OVHM cells were cultured in Dulbecco's modified Eagle's medium with high glucose. All cells were cultured with 10% heat‐inactivated fetal calf serum (FCS), 5% antimycotics and antibiotics in 5% CO_2_ at 37°C. The construction and the purification of adenoviruses were performed as described previously.^14^, ^15^, ^17^, ^18^

### 2.2 Cloning of EHMK cell lines

The EHMK cell line was established from human lung adenocarcinoma in Ehime University (Ehime, Japan) and cultured in RPMI with 10% heat‐inactivated FCS, 5% antimycotics and antibiotics in 5% CO_2_ at 37°C. EHMK cells were cloned by limiting dilution. EHMK cells were seeded at 0.25 cells/well in 96‐well plates and incubated in 100 μl/well of RPMI medium, with another 100 μl/well of RPMI medium added into each well every 1 or 2 weeks. We selected cells that had spread from an initial single cell/well and showed stable growth. These cells were cultured and analyzed as potential carrier cells.

### 2.3 Cytotoxic assay

To evaluate the cytotoxic effects of AdE3‐*midkine*‐infected cell clones, EHMK cells grown in 96‐well plates were replated in 5 × 10^6^ cells/well in six‐well plates, infected with AdE3‐*midkine* at 200 multiplicity of infection (MOI) for 3 h in RPMI medium without FCS and cultured overnight in RMPI medium with 10% FCS. The infected clones were trypsinized and added to HEY cells at 2000 cell/well in 96‐well plates with or without high titer (600×) of anti‐adenovirus antibodies (Takeda Pharmaceutical, Osaka, Japan) for 5 days. Cells were fixed and stained with 0.5% crystal violet in 20% ethanol and absorbance was measured by a spectrophotometer at 550 nm (*n* = 5). The cytotoxic effects of AdE3‐*midkine*‐infected clones against HEY cells were calculated from the 50% inhibition level of cell growth (IC_50_).

### 2.4 Establishment of subcutaneous and intraperitoneal ovarian tumor model in syngeneic mice and treatments

Murine OVHM cells (10^6^) were subcutaneously and intraperitoneally injected into female (C57BL/6 × C3H/He) F1 mice (CLEA Japan Inc., Tokyo, Japan) (*n* = 10 for each injected substance). In total, 140 mice (70 for subcutaneous tumors and 70 for intraperitoneal tumors) were randomized into seven treatment groups (*n* = 10 per group). A549 and EHMK‐51‐35 carrier cells were irradiated at 200 Gy, cryopreserved in liquid nitrogen and thawed before injection. Mice were treated as indicated: (i) phosphate‐buffered saline (PBS) alone; (ii) AdE3‐*midkine* at 10^10^ plaque‐forming units (PFU); (iii) AdE3‐*midkine* at 10^10^ PFU with Ad‐*mGM‐CSF* at 10^10^ PFU; (iv) A549 carrier cells (5 × 10^6^) infected with AdE3‐*midkine* at 20 MOI; (v) A549 carrier cells (5 × 10^6^) infected with both AdE3‐*midkine* at 20 MOI and Ad‐*mGM‐CSF* at 20 MOI; (vi) EHMK‐51‐35 carrier cells (5 × 10^6^) infected with AdE3‐*midkine* at 20 MOI; or (vii) EHMK‐51‐35 carrier cells (5 × 10^6^) infected with both AdE3‐*midkine* at 20 MOI and Ad‐*mGM‐CSF* at 20 MOI for 33 h each. Treatments were injected into subcutaneous tumors 5–8 mm in diameter on days 0, 1 and 2 and into intraperitoneal spaces on days 0, 2 and 4 after the intraperitoneal injection of OVHM cells on day −4. Intraperitoneal injections were diluted with 10 ml of PBS to spread the injections throughout the abdominal cavity. Mice were preimmunized with Ad‐*β‐gal* (1 × 10^10^ PFU) on day −14 before the OVHM subcutaneous and intraperitoneal injection.

### 2.5 Quantitative real‐time polymerase chain reaction (qPCR)

DNA was extracted from blood, saliva, feces, urine and whole organs using commercially available nucleic acid extraction kits and stored at −80°C until use. The nucleic acid extraction kits used were QlAmp DNA Mini Kit (plasma and organs), QlAmp DNA Blood Mini Kit (saliva), QlAmp Viral RNA Mini Kit (urine) and QlAmp DNA Stool Kit (feces) (Qiagen, Tokyo, Japan) and all extractions were performed in accordance with the manufacturer's instructions. Hemolysis was evaluated using the Combur Test^Ⓡ^ (Roche Diagnostics Co., Tokyo, Japan). Urine was centrifuged at 3000 rpm (1500 g) for 5 min, sterilized by filtering with 0.45 μm of Millex‐HV (Merck Co., Tokyo, Japan) and used for the analyses. The extraction method for each sample is shown in Table 1. qPCR was performed using the LightCycler® Nano (Roche Diagnostics, Tokyo, Japan) with 10 μl of MightyAmp (Takara, Kusatsu, Japan), 1 μl of forward primer, 1 μl of reverse primer and 8 μl of sample in the reaction mix. For the PCR, samples were denatured at 98°C for 2 min, followed by 40 cycles of denaturing at 98°C for 10 s, annealing at 64°C for 15 s and extending 68°C for 30 s, and, finally, melting at 60–95°C. Specific primers for wild‐type adenovirus (AdE3, replication competent wild‐type adenovirus), AdE3‐*midkine*, Ad‐*cGM‐CSF* and Ad‐*fGM‐CSF* are shown in Table 2.

**Table 1:** Extraction methods of each sample

Sample	Extraction method
Plasma	Directly used at 200 μl
Urine	200 μl of supernatant after centrifugation (9000 *g* for 10 min)
Sputum	Infiltrate 200 μl of sputum into a filter paper, dry and elute by 800 μl of PBS and use 200 μl
Stool	Suspend 200 mg in 800 μl PBS, centrifuge (9000 *g* for 10 min) and use 200 μl of supernatant,
Organs	Homogenize 200 mg

**Table 2:** Specific primers for each adenovirus in qPCR

Primer	Adenovirus vector		Sequence	Pair	Amplification number
YS14‐008	Wild‐type adenovirus (AdE3) 5′	F	CGGGTCAAAGTTGGCGTTTT	008/009	184 bp
YS14‐009	Wild‐type adenovirus (AdE3) 3′	R	CGGCTCGGAGGAGAAAACTC		
F49	AdE3‐*midkine* 5′	F	AGGTGTTTTCCGCGTTC	F49/R49	229 bp
R49	AdE3‐*midkine* 3′	R	CGTTCTCAGGCCTCAG		
YS14‐001	Ad‐*cGM‐CSF* 5′	F	CCATAGAAGACACCGGGACC	001/002	109 bp
YS14‐002	Ad‐*cGM‐CSF* 3′	R	TGCTTTATTCATCACAGCAGTCACG		
YS14‐001	Ad‐*fGM‐CSF* 5′	F	CCATAGAAGACACCGGGACC	001/003	109 bp
YS14‐003	Ad‐*fGM‐CSF* 3′	R	CGTTTCATTCATCACAGCAGTTATT		

F, forward primer, R, reverse primer.

### 2.6 Plaque assay

Human embryonic kidney‐derived cells (293 cells) were seeded at 2 × 10^4^ cells/well in a 96‐well plate and cultured in a Dulbecco's modified Eagle's medium high‐glucose culture medium supplemented with 10% FCS, 5% antibiotics and antimycotics at 37°C and 5% CO_2_ for 24 h. Plasma, feces, saliva, urine and systemic organ samples were serially diluted 10 times and cultured for 7 days with culture medium being added every 3 days. The median tissue culture infectious dose was calculated by checking cytopathic effects. Details of the plaque assay of adenoviruses have been described previously.^14^, ^15^, ^17^, ^18^

### 2.7 Acute toxicity test of EHMK‐51‐35 carrier cells infected with AdE3‐*midkine* and ad‐*cGM‐CSF* in beagle dogs

The experimental protocol of the animal studies was approved by the Institutional Animal Care and Use Committee at Ehime University and Tokyo University of Agriculture and Technology. EHMK‐51‐35 carrier cells were infected with AdE3‐*midkine* and Ad‐*cGM‐CSF* at 20 MOI for 33 h each and then 10^4^, 10^5^, 10^6^ or 10^7^ cells were injected subcutaneously into the thigh of four beagle dogs (*n* = 1 for each concentration) (Table 3). EHMK‐51‐35 carrier cells were irradiated at 200 Gy, cryopreserved in liquid nitrogen and thawed before injection. Body weight, clinical symptoms and body temperature were observed every day, and saliva, feces and urine were collected every day. Blood was collected before and 1, 3, 5, 7 and 14 days after injection (Figure 4A). Dogs were euthanized 14 days after injection and the organs of each dog were harvested (Table 4).

**Table 3:** Backgrounds of beagle dogs in acute toxicity test of EHMK‐51‐35 carrier cells infected with AdE3‐*midkine* and Ad‐*cGM‐CSF* in beagle dogs

Background				
No	Dose	Sex	Age	Body weight
1 (10–11)	10^4^ cells/head (2 × 10^5^ PFU)	Unfixed female	7 years old	10 kg
2 (10–01)	10^5^ cells/head (2 × 10^6^ PFU)	Unfixed male	1 year old	10 kg
3 (10–32)	10^6^ cells/head (2 × 10^7^ PFU)	Unfixed male	0.7 years old	10 kg
4 (05–06)	10^7^ cells/head (2 × 10^8^ PFU)	Unfixed female	1 year old	10 kg

**Table 4:** List of harvested organs in acute toxicity test of EHMK‐51‐35 carrier cells infected with AdE3‐*midkine* and Ad‐*cGM*‐*CSF* in beagle dogs

No.	Harvested organs	
1 (10–11)	Brain/heart/lung/liver/spleen/stomach/ kidney/bladder/salivary gland/ submandibular lymph node/tonsilla	Uterus/ovary
2 (10–01)	Brain/heart/lung/liver/spleen/stomach/ kidney/bladder/salivary gland/ submandibular lymph node/tonsilla	Prostate/testis
3 (10–32)	Brain/heart/lung/liver/spleen/stomach/ kidney/bladder/salivary gland/ submandibular lymph node/tonsilla	Prostate/testis
4 (05–06)	Brain/heart/lung/liver/spleen/stomach/ kidney/bladder/salivary gland/ submandibular lymph node/tonsilla	Uterus/ovary

### 2.8 Acute toxicity test of AdE3‐*midkine* in beagle dogs

The experimental protocol of animal studies was approved by the Institutional Animal Care and Use Committee at Ehime University and Tokyo University of Agriculture and Technology. AdE3‐*midkine* at 10^10^ PFU was injected subcutaneously into three beagle dogs (Table 5). Body weight, clinical symptoms, body temperature, and saliva, urine and blood of the first beagle dog (No. 5) were observed and collected before and at 0, 3, 6, 12 and 24 h after injection (Figure 5A). Feces were collected before and 24 h after injection. The dog was euthanized 24 h after injection and the organs were harvested (Table 6). Body weight, clinical symptoms and body temperature of the second dog (No. 6) were observed every day, and saliva, feces, urine and blood were collected before and every day after injection (Figure 5A). The dog was euthanized 4 days after injection and the organs were harvested (Table 6). Body weight, clinical symptoms and body temperature of the third dog (No. 7) were observed every day; saliva, feces, urine and blood were collected before and 0, 1, 2, 3, 4, 5, 7, 9 and 11 days after injection (Figure 5A). The dog was euthanized 11 days after injection and the organs were harvested (Table 6). Harvested organ tissues of each dog were subjected to histopathological and qPCR analysis.

**Table 5:** Backgrounds of beagle dogs in acute toxicity test of AdE3‐*midkine* in beagle dogs

Background					
No.	Dose (PFU)	Sex	Age	Body weight	Sacrificed date
5 (12‐08C)	10^10^	Unfixed female	8 months old	7 kg	24 hours
6 (12‐18B)	10^10^	Unfixed male	8 months old	5.5 kg	Day 4
7 (12‐12A)	10^10^	Unfixed male	8 months old	7 kg	Day 11

### 2.9 Chronic toxicity test of EHMK‐51‐35 carrier cells infected with AdE3‐*midkine* in rabbits

The experimental protocol of animal studies was approved by the Institutional Animal Care and Use Committee at Ehime University and Tokyo University of Agriculture and Technology. EHMK‐51‐35 carrier cells were infected with AdE3‐*midkine* at 20 MOI for 33 h and then 10^7^ cells were injected five times into VX2 tumors in the thighs of two rabbits (SLC:JW/CSK; Japan SLC Inc., Shizuoka, Japan). A non‐injected tumor‐bearing rabbit served as control. EHMK‐51‐35 carrier cells were irradiated at 200 Gy, cryopreserved in liquid nitrogen and thawed before injection. Body weight, clinical symptoms and body temperature were observed every day, and blood was collected until rabbits were euthanized 11 or 14 days after the start of injection. Organs of each rabbit were harvested whole and subjected to histological examination.

### 2.10 Statistical analysis

Data are expressed as the mean ± SD and were analyzed using an unpaired *t*‐test, the Welch tests, one‐way analysis of variance and the Kruskal–Wallis test. Survival data were analyzed with the generalized Wilcoxon tests. *P <* 0.05 was considered statistically significant.

## 3 RESULTS

### 3.1 Cloning of carrier cells

**Figure 1:**
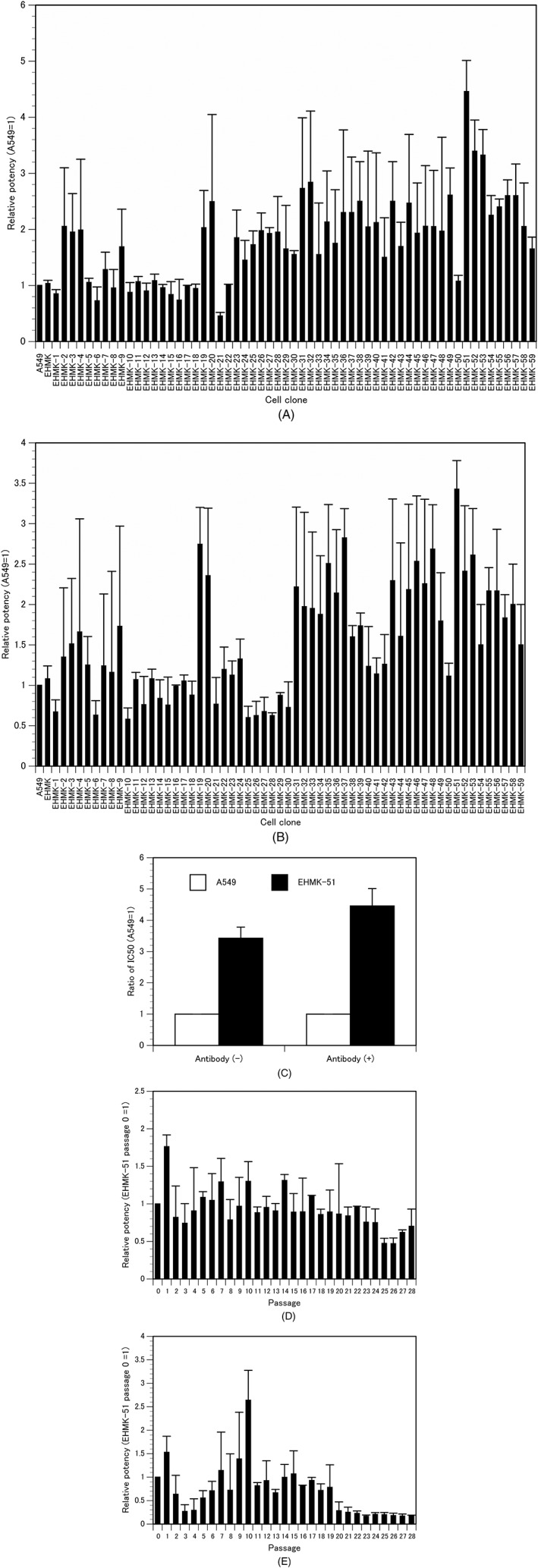
Cloning of EHMK carrier cells. Relative *in vitro* antitumor activity of AdE3‐*midkine*‐infected cloned EHMK carrier cells in ovarian cancer HEY cells (A) with or (B) without anti‐adenovirus antibodies; IC_50_ of A549 carrier cells = 1. Bars indicate the SD. (C) Relative *in vitro* antitumor activity of EHMK‐51 carrier cells on A549 carrier cells with or without anti‐adenovirus antibodies; IC_50_ of A549 carrier cells = 1. *In vitro* antitumor activity at each passage of cloned EHMK‐51 carrier cells (D) with or (E) without anti‐adenovirus antibodies; IC_50_ of EHMK‐51 carrier cells at passage 0 = 1

To clone a new carrier cell with significant antitumor effect, we cloned cells from EHMK cells by limiting dilution and examined the antitumor activity of each cell clone infected with AdE3‐*midkine* in HEY ovarian cancer cells. The *in vitro* antitumor activity was evaluated by comparing the IC_50_ with that of A549 carrier cells infected with AdE3‐*midkine* with or without anti‐adenovirus antibodies set as 1. Our established EHMK carrier cells infected with AdE3‐*midkine* had a similar IC_50_ value as A549 carrier cells with or without anti‐adenovirus antibodies (Figure 1A, B). The antitumor activities of the cloned EHMK‐51 carrier cells were 4.46‐ or 3.43‐fold higher than A549 carrier cells with or without anti‐adenovirus antibodies, respectively (*p* < 0.05) (Figure 1A–C). To investigate the stability of the antitumor activity of the cloned EHMK‐51 carrier cells, the antitumor activity of each passage of EHMK‐51 carrier cells was compared with the IC_50_ of the original EHMK‐51 carrier cells infected with AdE3‐*midkine* set as 1. The results showed that the antitumor activity decreased after 25 passages or 20 passages with or without anti‐adenovirus antibodies, respectively (Figure 1D and E).

**Figure 2:**
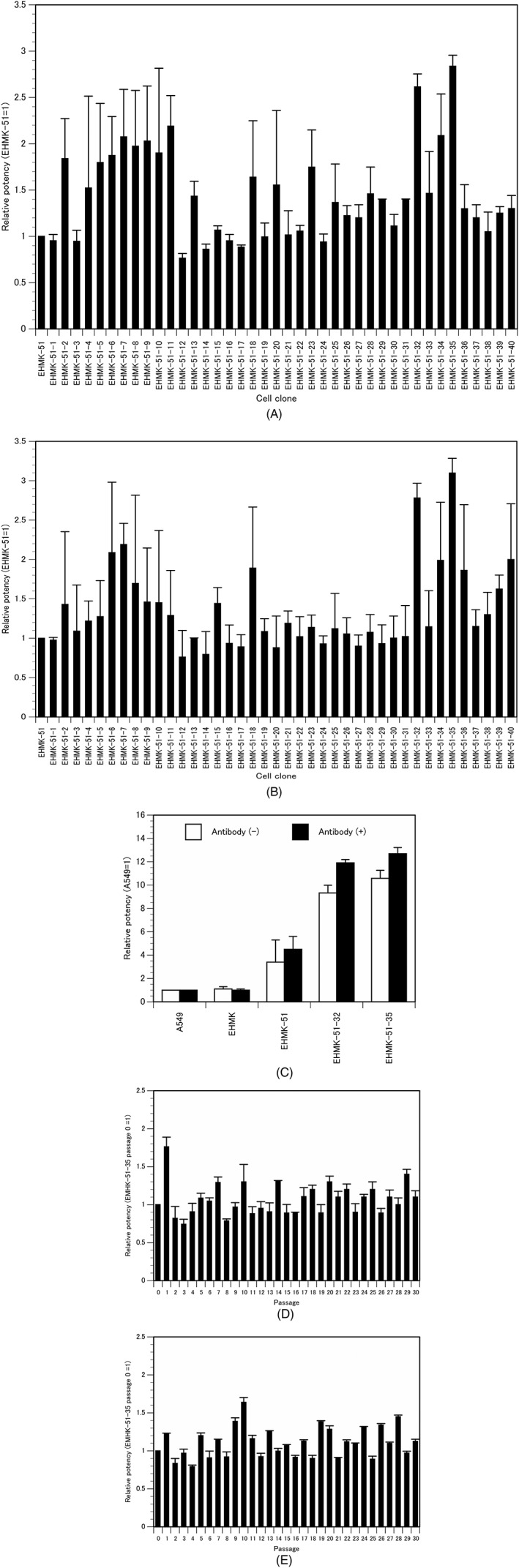
Cloning of EHMK‐51 carrier cells. Relative *in vitro* antitumor activity of AdE3‐*midkine*‐infected cloned EHMK‐51 carrier cells in ovarian cancer HEY cells (A) with or (B) without anti‐adenovirus antibodies; IC_50_ of EHMK‐51 carrier cells = 1. Bars indicate the SD. (C) Relative *in vitro* antitumor activity of EHMK‐51 carrier cells on A549 carrier cells with or without anti‐adenovirus antibodies; IC_50_ of A549 carrier cells = 1. Bars indicate the SD. *In vitro* antitumor activity at each passage of cloned EHMK‐51 carrier cells (D) with or (E) without anti‐adenovirus antibodies; IC_50_ of EHMK‐51‐35 carrier cells at passage 0 = 1

To clone new carrier cells with stable antitumor activity from the EHMK‐51 carrier cells, the EHMK‐51 cells were further cloned by limiting dilution. The *in vitro* antitumor activity of each cell clone infected with AdE3‐*midkine* was compared with the IC_50_ of the original EHMK‐51 carrier cells infected with AdE3‐*midkine*. The antitumor activities of the EHMK‐51‐32 and EHMK‐51‐35 carrier cell clones were 2.61‐ and 2.84‐fold higher than that of EHMK‐51 carrier cells with anti‐adenovirus antibodies and 2.78‐ and 3.10‐fold higher than EHMK‐51 carrier cells without anti‐adenovirus antibodies, respectively (both *p* < 0.05) (Figure 2A and B). The antitumor activities of EHMK‐51‐32 and EHMK‐51‐35 carrier cell clones were 11.91‐ and 12.69‐fold higher than A549 carrier cells with anti‐adenovirus antibodies and 9.33‐ and 10.58‐fold higher than A549 carrier cells without anti‐adenovirus antibodies, respectively (both *p* < 0.05) (Figure 2C). The antitumor activities of EHMK‐51‐35 and EHMK‐51‐35 carrier cell clones did not decrease within 30 passages with or without anti‐adenovirus antibodies (Figure 2D and E). Because EHMK‐51‐35 carrier cells demonstrated the greatest antitumor effect among the tested clones and an even greater effect with anti‐adenovirus antibodies compared to that without ant‐adenovirus antibodies, we selected these as carrier cells for the *in vivo* experiments.

### 3.2 Antitumor effect of EHMK‐51‐35 carrier cells on subcutaneous tumor‐bearing mice

**Figure 3:**
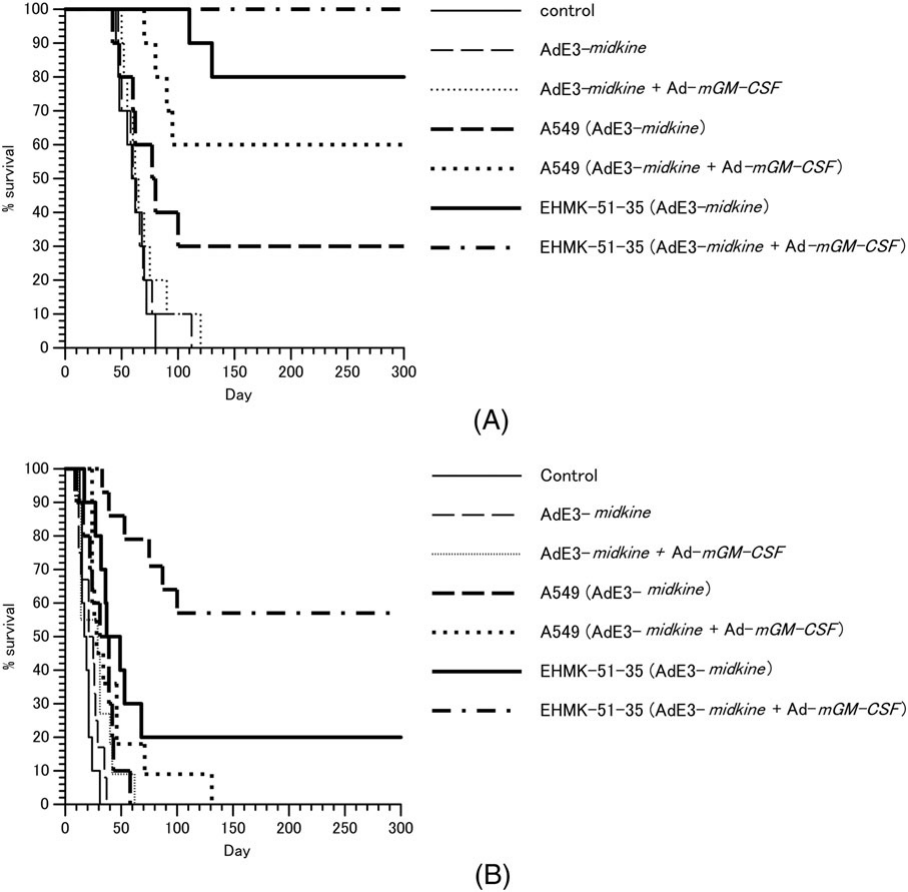
Antitumor effect of EHMK‐51‐35 carrier cells in syngeneic mouse model. EHMK51–35 carrier cells infected with AdE3‐*midkine* and ad‐*mGM‐CSF* were injected three times into (A) OVHM ovarian cancer subcutaneous tumors and (B) intraperitoneal disseminated tumors after pre‐immunization of adenovirus. Results are shown as a Kaplan–Meier survival curve

We next examined the antitumor effect of EHMK‐51‐35 carrier cells in subcutaneous OVHM ovarian tumors in syngeneic mice. EHMK‐51‐35 carrier cells infected with AdE3‐*midkine* or co‐infected with AdE3‐*mikine* and Ad‐*mGM‐CSF* were injected three times into subcutaneous OVHM ovarian tumors in syngeneic mice. The Kaplan–Meier method was used to compare survival rates with those of mice injected with A549 carrier cells infected with AdE3 ‐*midkine* or co‐infected with AdE3‐*midkine* and Ad‐*mGM‐CSF*. Three subcutaneous intratumoral injections of adenovirus alone, AdE3‐*midkine*, or AdE3‐*midkine* with Ad‐*cGM‐CSF* showed no significant antitumor effect compared to controls (Figure 3A). A549 carrier cells infected with AdE3‐*midkine* or co‐infected with AdE3‐*midkine* and Ad‐*mGM‐CSF* induced 30% or 60% of complete tumor reduction, respectively (*p <* 0.05). Notably, EHMK‐51‐35 carrier cells infected with AdE3‐*midkine* or co‐infected with AdE3‐*midkine* and Ad‐*mGM‐CSF* induced 80% or 100% of complete tumor reduction, respectively, which increased the survival rates significantly compared to A549 carrier cells (*p <* 0.05).

### 3.3 Antitumor effect of EHMK‐51‐35 carrier cells on intraperitoneal tumor‐bearing mice

We performed similar analyses on the antitumor effect of EHMK‐51‐35 carrier cells in intraperitoneal OVHM ovarian tumors in syngeneic mice. Three intraperitoneal injections with adenovirus alone of AdE3‐*midkine* or AdE3‐*midkine* with Ad‐*cGM‐CSF* showed no significant antitumor effect compared to controls (Figure 3B). In addition, A549 carrier cells infected with AdE3‐*midkine* or co‐infected with AdE3‐*midkine* and Ad‐*mGM‐CSF* also showed no significant antitumor effect compared to controls. However, EHMK‐51‐35 carrier cells infected with AdE3‐*midkine* or co‐infected with AdE3‐*midkine* and Ad‐*mGM‐CSF* induced 20% or 60% of complete tumor reduction, respectively, which increased the survival rates significantly compared to controls and A549 carrier cells (*p <* 0.05).

### 3.4 Acute toxicity test of carrier cells infected with AdE3‐*midkine*‐ and ad‐*cGM‐CSF* in beagle dogs

**Figure 4:**
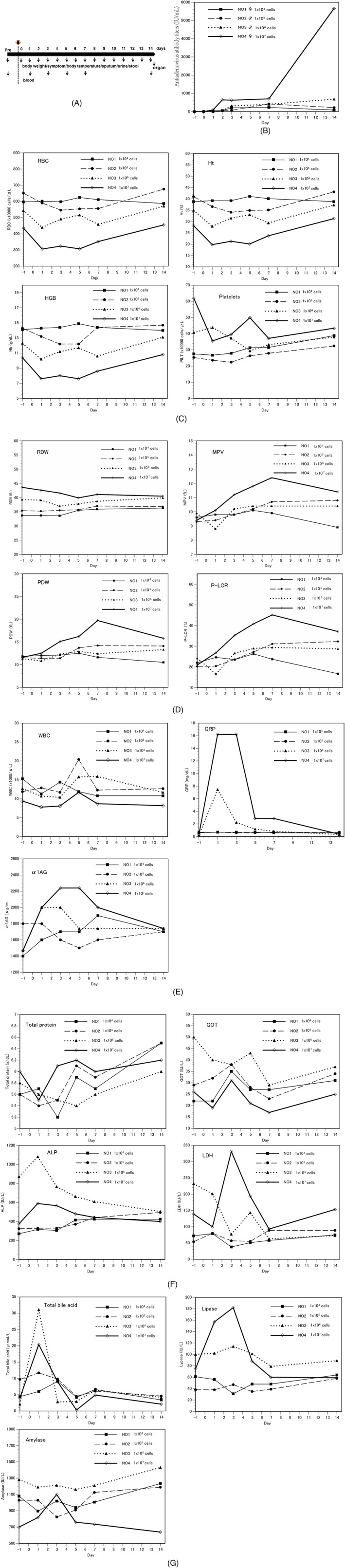
Blood analysis results in acute toxicity test of single injections of EHMK‐51‐35 carrier cells infected with AdE3‐*midkine* and ad‐*cGM‐CSF* in beagle dogs. (A) Protocol for injections of EHMK‐51‐35 carrier cells in acute toxicity test of EHMK‐51‐35 carrier cells infected with AdE3‐*midkine* and Ad‐*cGM*‐*CSF* in beagle dogs, (B) Anti‐adenovirus antibody titers, (C, D) CBC, (E) acute inflammation reaction and (F, G) blood chemistry results

To evaluate the acute safety of AdE3‐*midkine*‐ and Ad‐*cGM‐CSF*‐infected carrier cells, single doses of dose‐escalated carrier cells were injected subcutaneously into four beagle dogs and the dogs were sacrificed 14 days later. Organs were removed for histological examination and qPCR of AdE3‐*midkine* and Ad‐*cGM‐CSF*, and adenovirus bioactivity was evaluated (plaque assay) (Table 4). Anti‐adenovirus antibody titers began to rise 2 days after carrier cell injections and increased by 86×, 218×, 681× and 5665× at day 14 after injections of 10^4^ cells, 10^5^ cells, 10^6^ cells and 10^7^ cells, respectively (Figure 4B). Red blood cell (RBC), hematocrit (Ht), hemoglobin (HGB) and platelets decreased over 1–5 days after injection of 10^7^ carrier cells (Figure 4C). Mean platelet volume (MPV), platelet distribution width (PDW) and platelet large cell ratio (P‐LCR) increased during days 1–14 after injection of 10^7^ carrier cells (Figure 4D). In the acute inflammation test, C‐reactive protein (CRP) and α1‐acid glycoprotein (α1AG) increased over the first 7 days after injections of 10^6^ and 10^7^ carrier cells (Figure 4E). In blood biochemistry tests, alkaline phosphatase (ALP) and lactate dehydrogenase (LDH) increased during days 3–5 after injection of 10^7^ carrier cells (Figure 4F). Total bile acid increased on day 1 after injection of 10^6^ and 10^7^ carrier cells. Amylase and lipase increased during days 1–3 after injection of 10^7^ carrier cells (Figure 4G). Daily activities and appetite decreased on days 1 to 2 in two dogs after injections of 10^6^ and 10^7^ carrier cells but improved after several days. The dogs injected with 10^4^ and 10^5^ carrier cells showed no abnormal clinical symptoms. A summary of abnormal clinical laboratory findings of the acute toxicity test of carrier cells in beagle dogs is shown in Table 7.

**Table 6:** List of harvested organs in acute toxicity test of AdE3‐*midkine* in beagle dogs

No	Harvested organs	
5 (12‐08C)	Brain/heart/lung/liver/spleen/stomach/kidney/bladder/salivary gland/submandibular lymph node/tonsilla	Uterus/ovary
6 (12‐18B)	Brain/heart/lung/liver/spleen/stomach/kidney/bladder/salivary gland/submandibular lymph node/tonsilla	Prostate/testis
7 (12‐12A)	Brain/heart/lung/liver/spleen/stomach/kidney/bladder/salivary gland/submandibular lymph node/tonsilla	Prostate/testis

### 3.5 qPCR and plaque assay

We constructed primer sets for each adenovirus (see Supporting information, Figure S1A) and performed qPCR to determine specificity. AdE3‐*midkine* was amplified by qPCR in 10 to 10^8^ PFU in a dose‐dependent manner with a specific melting curve (see Supporting information, Figure S1B). qPCR of 10^8^ PFU of AdE3, AdE3‐*midkine*, Ad‐*cGM‐CSF* and Ad‐*fGM‐CSF*, using specific primers for AdE3‐*midkine*, amplified only AdE3‐*midkine* with a specific melting curve for AdE3‐*midkine* (see Supporting information, Figure S1C). Similarly, qPCR with specific primers successfully amplified AdE3, Ad‐*cGM‐CSF* and Ad‐*fGM‐CSF* with specific melting curves for each adenovirus (see Supporting information, Figure S1D–F).

We detected 6.96 and 16.4 copies/ml of AdE3‐*midkine* in blood samples from dogs at 1 and 3 days after injection of 10^7^ carrier cells, respectively, and 26.2 and 54.3 copies/ml of AdE3‐*midkine* in blood samples from dogs at 1 and 3 days after injection of 10^6^ carrier cells, respectively, although not in other samples from the four dogs. Plaque assays showed no significant adenovirus activity in any of the samples from the dogs including blood. Ad‐*cGM‐CSF* DNA was not detected in any samples from the four dogs.

AdE3‐*midkine* DNA and biologically active adenovirus (plaque assay) were not detected in blood, feces, urine, saliva or removed organ tissues in the three dogs injected with AdE3‐*midkine* in the acute toxicity test.

### 3.6 Acute toxicity test of AdE3‐*midkine* in beagle dogs

**Figure 5:**
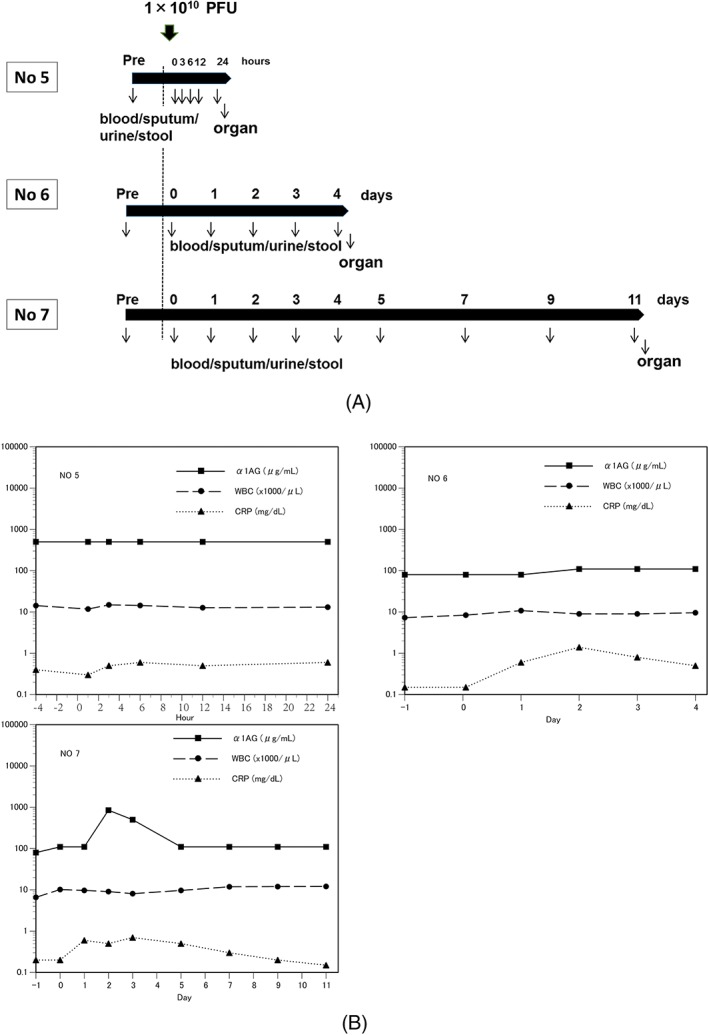
(A) Protocol for injections of AdE3‐*midkine* in acute toxicity test of AdE3‐*midkine* in beagle dogs, (B) Result of acute inflammation reaction in acute toxicity test in beagle dogs with single injections of AdE3‐*midkine*

To evaluate acute safety of AdE3‐*midkine*, 10^10^ PFU of AdE3‐*midkine* was injected subcutaneously into the thighs of three beagle dogs. Dogs were sacrificed at 24 h, 4 days or 11 days later; organs were removed for histological examination and qPCR was performed for AdE3‐*midkine* (Table 6). Although no change was observed in white blood cell (WBC) levels, α1AG increased over days 2–4 after injections (Figure 5B). CRP started to increase 3 h after injection of AdE3‐*midkine,* peaked on day 2 and returned to normal on day 9 (Figure 5B). Other blood tests did not change after injections (see Supporting information, Figure S2). No abnormal clinical symptoms were detected in any of the three beagle dogs. A summary of abnormal clinical laboratory findings of the acute toxicity test of AdE3‐*midkine* in beagle dogs is shown in Table 8.

**Table 7:** Summary of abnormal clinical laboratory findings in acute toxicity test of EHMK‐51‐35 carrier cells infected with AdE3‐*midkine* and Ad‐*cGM*‐*CSF* in beagle dogs

Clinical laboratory findings				
	1 × 10^4^ cells	1 × 10^5^ cells	1 × 10^6^ cells	1 × 10^7^ cells
Increased	Antibodies,	Antibodies,	Antibodies, CRP, α1AG, Total bile acid,	Antibodies, MPV, PDW, P‐LCR, CRP, α1AG, ALP, LDH, Total bile acid, lipase, amylase
Decreased	–	–	Daily activities	Daily activities, RBC, Ht, HGB, PLT

**Table 8:** Summary of abnormal clinical laboratory findings in acute toxicity test of AdE3‐*midkine* in beagle dogs

Clinical laboratory findings			
	24 hours	4 days	11 days
Increased	CRP	CRP, α1AG	CRP, α1AG
Decreased	–	–	–

### 3.7 Chronic toxicity test of EHMK‐51‐35 carrier cells infected with AdE3‐*midkine* in rabbits

**Figure 6:**
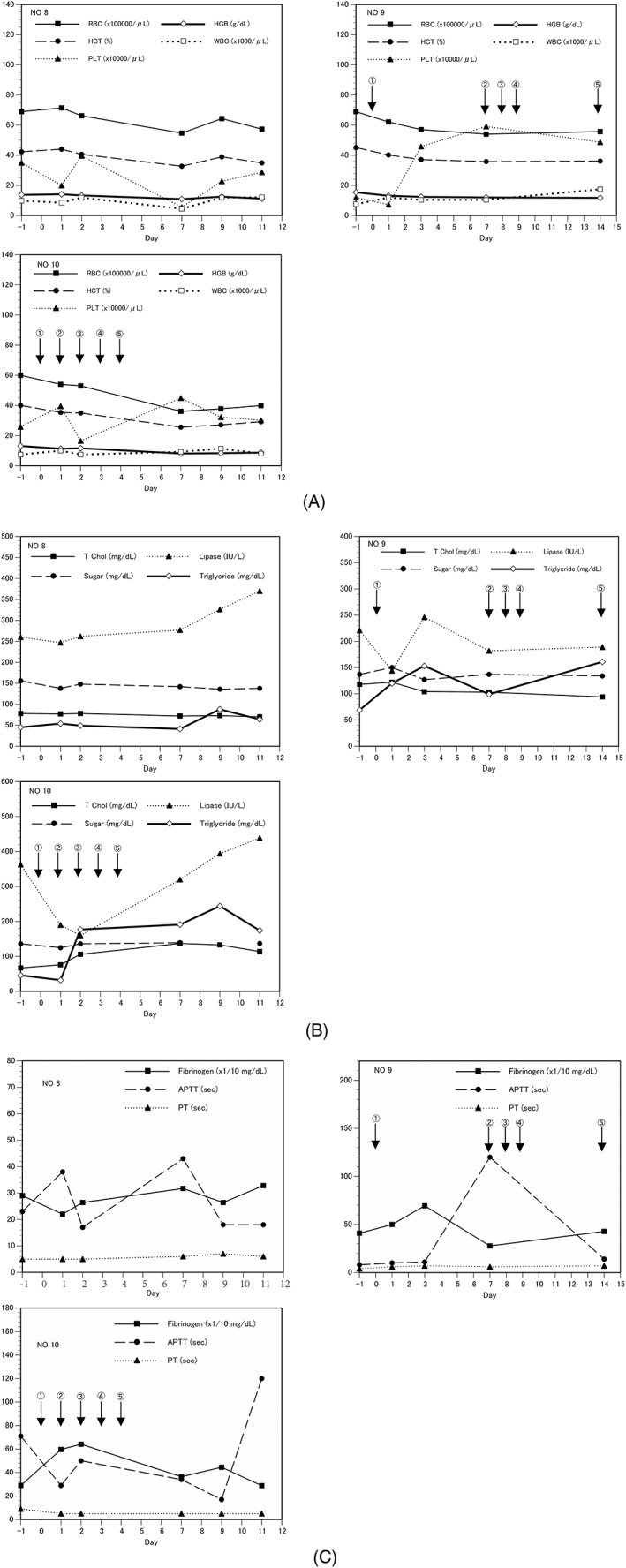
Blood analysis results in chronic toxicity test of EHMK‐51‐35 carrier cells infected with AdE3‐*midkine* in rabbits harboring VX2 tumors. Five intratumoral injections of carrier cells were performed. (A) CBC, (B) blood chemistry and (C) hemostatic function test results

To evaluate the safety of carrier cells infected with AdE3‐*midkine* in tumor‐bearing animals, 10^7^ EHMK‐51‐35 carrier cells infected with AdE3‐*midkine* were injected five times into VX2 tumors of two rabbits. RBC, Ht and HGB decreased, and WBC, platelets, triglyceride and activated partial thromboplastin time (APTT) increased in the injected rabbits compared to controls (Figure 6), whereas other blood tests showed no changes (see Supporting information, Figure S3). Both experimental rabbits showed decreased motility and appetite after injections, although no other serious side effects were observed. Histopathological findings showed no significant abnormalities except for inflammatory changes of liver and jejunum. A summary of the abnormal clinical laboratory findings of the chronic toxicity test of carrier cells in rabbits is provided in Table 9.

**Table 9:** Summary of abnormal clinical laboratory findings in chronic toxicity test of five injections of EHMK‐51‐35 carrier cells infected with AdE3‐*midkine* in rabbits

	Five injections
Increased	PLT, WBC, triglyceride, APTT
Decreased	RBC, Ht, HGB, daily activities

## 4 DISCUSSION

The newly cloned EHMK‐51‐35 carrier cells in the preent study showed approximately 12‐ or 10‐fold higher *in vitro* antitumor effects with or without anti‐adenovirus antibodies, respectively, compared to A549 carrier cells. A549 carrier cells were previously shown to inhibit tumor growth via induction of the anti‐adenoviral CTL response and further anti‐tumor immunity only after pre‐immunization with adenovirus, as demonstrated by the CTL assay.^14^ A similar immune reaction might be induced with the newly developed carrier cells because EHMK‐51‐35 carrier cells also inhibited tumor growth after pre‐immunization with adenovirus. The greater *in vitro* antitumor effect of EHMK‐51‐35 carrier cells compared to A549 carrier cells might be a result of the increased production of oncolytic adenovirus. The greater *in vitro* antitumor effect in the presence of anti‐adenovirus antibodies than without these antibodies might be a result of the more efficient non‐adenovirus receptor‐mediated infection by adenovirus‐containing cell fragments formed after cell lysis by oncolytic adenovirus.^14^ In the present study, EHMK‐51‐35 carrier cells infected with AdE3‐*midkine* and Ad‐*cGM‐CSF* showed 100% complete tumor reduction of subcutaneous mouse ovarian tumors and 60% complete tumor reduction of intraperitoneally metastasized mouse ovarian tumors. By contrast, A549 carrier cells infected with both AdE3‐*midkine* and Ad‐*cGM‐CSF* produced 60% and 0% of tumor regression, respectively. These results demonstrate significantly high antitumor effects of the EHMK‐51‐35 carrier cells, especially in the intraperitoneal tumors, compared to A549 carrier cells. This greater *in vivo* antitumor effect of EHMK carrier cells might be a result of further significant induction of anti‐adenoviral CTL and anti‐tumor immunity via the combination of GM‐CSF with more antigen‐presenting ability in addition to a greater direct antitumor effect via the increased oncolytic adenovirus production and infectivity.

Ovarian cancer easily disseminates into the intraperitoneal space, which greatly contributes to its poor prognosis. Therefore, improving the treatment outcome for intraperitoneal disseminated metastasis may improve the prognosis of ovarian cancer. More than 30 clinical trials of gene therapy for ovarian cancer have been conducted in the USA, although none have shown clinical efficacy.^1^ The present study shows promising results that could form the basis of a clinical trial for EHMK‐51‐35 carrier cells for ovarian cancer in the near future. Because digestive cancers, such as those of the pancreas, colon and stomach, also lead to intraperitoneal disseminated metastasis, EHMK‐51‐35 carrier cell therapy might also be effective for these tumors.

A small number of AdE3‐*midkine* DNA copies were detected in blood of beagle dogs on days 1–3 after injections of 10^6^ and 10^7^ carrier cells infected with AdE3‐*midkine* and Ad‐*cGM‐CSF*, although no adenovirus activity was found. AdE3‐*midkine* or biologically active adenovirus was not detected in any blood, stool, urine, saliva or removed tissue specimens other than these blood samples in beagle dogs after injections of carrier cells. The AdE3‐*midkine* DNA was not detected in any samples in beagle dogs after injections of AdE3‐*midkine*. AdE3‐*midkine* DNA detected by qPCR might comprise non‐infectious DNA fragments, which are degraded in the injected tissue and released into the blood because they have no biological activity. This suggests that treatment with oncolytic adenovirus or carrier cells would not require sequestration in P2A laboratories of isolated hospitalization facilities and could be administered on an outpatient basis.

Because CRP started to rise more than 3 h after injection of AdE3‐*midkine*, whereas α1AG and WBC did not rise, we consider CRP to be a more sensitive inflammatory response marker than α1AG and WBC. In dogs and cats with inflammation, infections, trauma and malignancies, α1AG increases and is high in pyometra and parvovirus enteritis in dogs and cats and in distemper and lymphomas in dogs. However, CRP was found to be superior to α1AG and WBC as an inflammatory reaction marker after injection of adenovirus in dogs.

Platelets decreased, whereas P‐LCR, MPV and PDW increased, after the high‐dose injection (10^7^ cells) of carrier cells into beagle dogs. However, platelets, P‐LCR, MPV and PDW increased over days 5–14 after injections of lower doses (10^4^ and 10^5^ cells) of carrier cells. Because disseminated intravascular coagulation (DIC) was mild, platelets might not be consumed and increased reactively; this is consistent with our previous results obtained in a rabbit safety test in which platelets increased after low‐dose injections of A549 carrier cells and decreased after high‐dose injections.^15^ Increased P‐LCR, MPV and PDW were considered to reflect an increased platelet production response. We found that RBC, Ht and HGB did not decrease after injections of AdE3‐*midkine* alone but decreased in a dose‐dependent manner after carrier cell injections into beagle dogs and decreased in rabbits whose tumors were injected five times. APTT was extended in rabbits treated with carrier cells, probably because DIC was caused by cytolysis of the injected carrier cells. These results are also consistent with our previous safety testing results in rabbits treated with A549 carrier cells.^15^ Almost no blood chemistry abnormality was detected after injection of AdE3‐*midkine* alone. No electrolyte abnormalities were detected after carrier cell injections and no abnormal values were detected in liver function or pancreatic function of beagle dogs that received high‐dose carrier cells. These results are consistent with our previous safety testing results in rabbits treated with A549 carrier cells.^15^

We observed decreased daily activity in beagle dogs treated with the highest dose and in tumor‐bearing rabbits injected five times. Because injected tumor‐bearing rabbits had relatively minor changes in clinical symptoms and laboratory findings compared to non‐tumor bearing dogs that received the highest‐dose of carrier cells, the changes of clinical symptoms and laboratory findings might be even smaller upon administration of carrier cells to dogs or cats with cancer. These animals might therefore be able to accept the highest dose of carrier cells safely. Dose limiting toxicity in carrier cell therapy was a decrease in daily activity and anemia as a result of RBC, HT and HGB reduction. These side effects are considered to be a result of DIC by cell lysis of carrier cells; it appears that the nuclear membrane was already broken at the time of freeze–thaw and the carrier cells were not alive before administration.^15^ By reducing the infectious dose and infection time of AdE3‐*midkine* and Ad‐*GM‐CSF* or by using fresh carrier cells without freezing, it may be possible to administer living carrier cells, thereby reducing side effects and increasing the dose.

With respect to future studies, after obtaining approval from the Ministry of Agriculture, Forestry and Fisheries of Japan, we plan to perform carrier cell therapy for cancers of dogs and cats and examine its safety and effectiveness. These results will inform the potential start of human clinical trials for refractory solid cancers. Because anti‐PD‐1 antibody^19^, ^20^, ^21^, ^22^ and anti‐CTLA4 antibody^23^ that suppress suppressor T cells can be formulated and effectively used in human cancers and might markedly activate carrier cell‐induced CTLs, we expect that the antitumor effect of carrier cell therapy could be further increased by combination with these preparations.

## CONFLICT OF INTEREST STATEMENT

The authors declare that they have no conflicts of interest.

## Supporting information

Figure S1. Quantitative real‐time PCR (qPCR) in an acute toxicity test in beagle dogs with single injections of EHMK‐51‐35 carrier cells infected with AdE3‐*midkine* and Ad‐*cGM‐CSF*. (A) Specific primers for AdE3‐*midkine*, AdE3, Ad‐*cGM‐CSF* and Ad‐*fGM‐CSF* in qPCR. Amplification and melting curves of qPCR. (B) 10 to 10^8^ copies of AdE3‐*midkine* with AdE3‐*midkine*‐specific primers. (C) 10^8^ copies of AdE3‐*midkine*, AdE3, Ad‐*cGM‐CSF* and Ad‐*fGM‐CSF* with AdE3‐*midkine*‐specific primers. (D) 10^8^ copies of AdE3‐*midkine*, AdE3, Ad‐*cGM‐CSF* and Ad‐*fGM‐CSF* with AE3‐specific primers. (E) 10^8^ copies of AdE3‐*midkine*, AdE3, Ad‐*cGM‐CSF* and Ad‐*fGM‐CSF* with Ad‐*cGM‐CSF*‐specific primers. (F) 10^8^ copies of AdE3‐*midkine*, AdE3, Ad‐*cGM‐CSF* and Ad‐*fGM‐CSF* with Ad‐*fGM‐CSF*‐specific primers.
Figure S2. Blood analysis results in an acute toxicity test in beagle dogs with single injections of AdE3‐*midkine*. (A, B) Complete blood count. (C–E) blood chemistry. (F) hemostatic function test results.
Figure S3. (A, B) Blood chemistry analysis in chronic toxicity test of EHMK‐51‐35 carrier cells infected with AdE3‐*midkine* in rabbits with VX2 tumors. Five intratumoral injections were performed.
