# Supplementary figures and images for: Cloning of carrier cells infected with oncolytic adenovirus driven by midkine promoter and biosafety studies
Source: J Gene Med. 2019 Feb 1;21(2-3):e3064. doi: 10.1002/jgm.3064 (PMC6590659; doi:10.1002/jgm.3064)

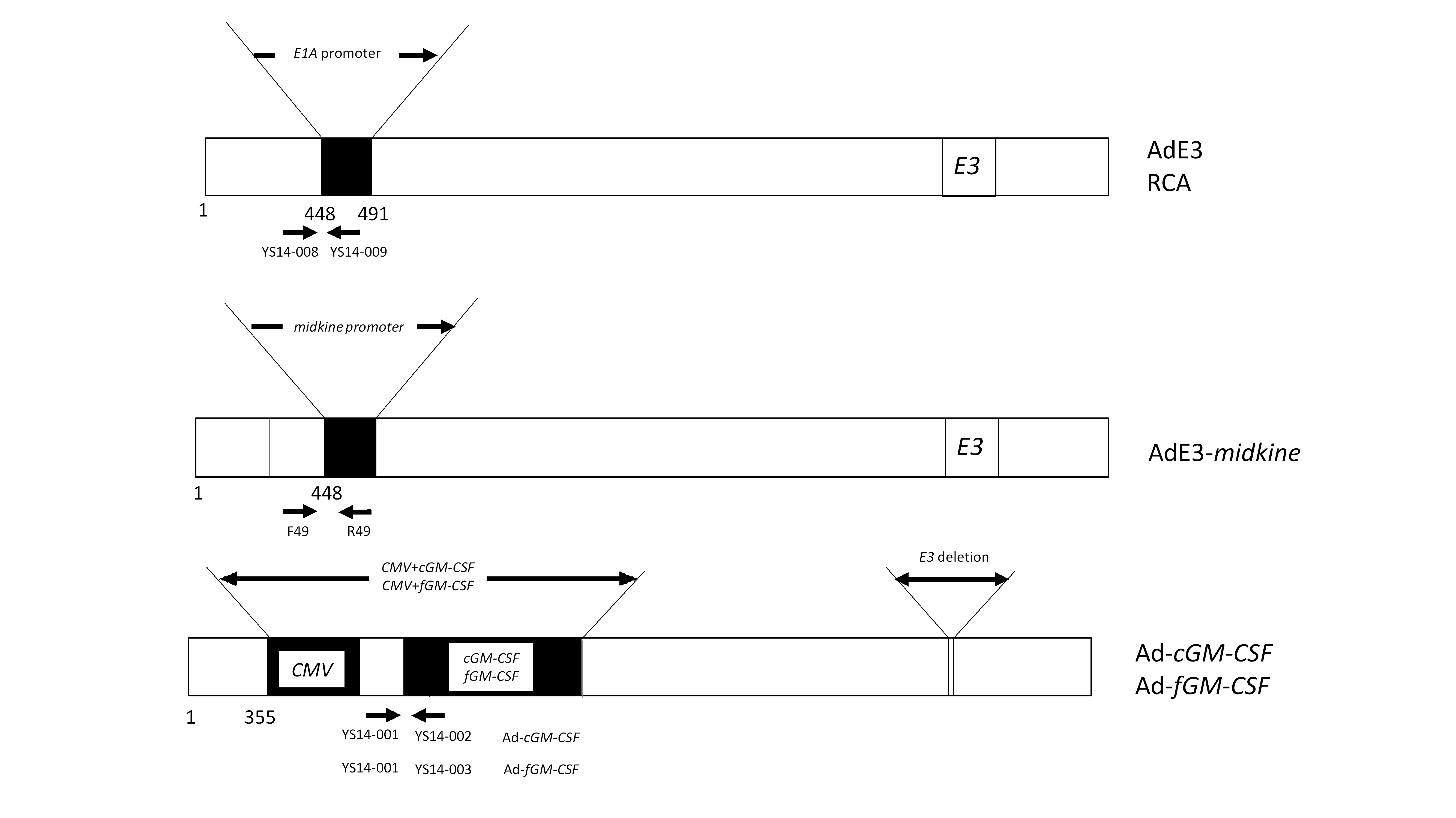

Supplement: Supplementary file 1 — Figure S1. Quantitative real‐time PCR (qPCR) in an acute toxicity test in beagle dogs with single injections of EHMK‐51‐35 carrier cells infected with AdE3‐midkine and Ad‐cGM‐CSF. (A) Specific primers for AdE3‐midkine, AdE3, Ad‐cGM‐CSF and Ad‐fGM‐CSF in qPCR. Amplification and melting curves of qPCR. (B) 10 to 108 copies of AdE3‐midkine with AdE3‐midkine‐specific primers. (C) 108 copies of AdE3‐midkine, AdE3, Ad‐cGM‐CSF and Ad‐fGM‐CSF with AdE3‐midkine‐specific primers. (D) 108 copies of AdE3‐midkine, AdE3, Ad‐cGM‐CSF and Ad‐fGM‐CSF with AE3‐specific primers. (E) 108 copies of AdE3‐midkine, AdE3, Ad‐cGM‐CSF and Ad‐fGM‐CSF with Ad‐cGM‐CSF‐specific primers. (F) 108 copies of AdE3‐midkine, AdE3, Ad‐cGM‐CSF and Ad‐fGM‐CSF with Ad‐fGM‐CSF‐specific primers. Figure S2. Blood analysis results in an acute toxicity test in beagle dogs with single injections of AdE3‐midkine. (A, B) Complete blood count. (C–E) blood chemistry. (F) hemostatic function test results. Figure S3. (A, B) Blood chemistry analysis in chronic toxicity test of EHMK‐51‐35 carrier cells infected with AdE3‐midkine in rabbits with VX2 tumors. Five intratumoral injections were performed. [file JGM-21-na-s001.zip › JGM3064-sup-0001-Fig S1A.tif]

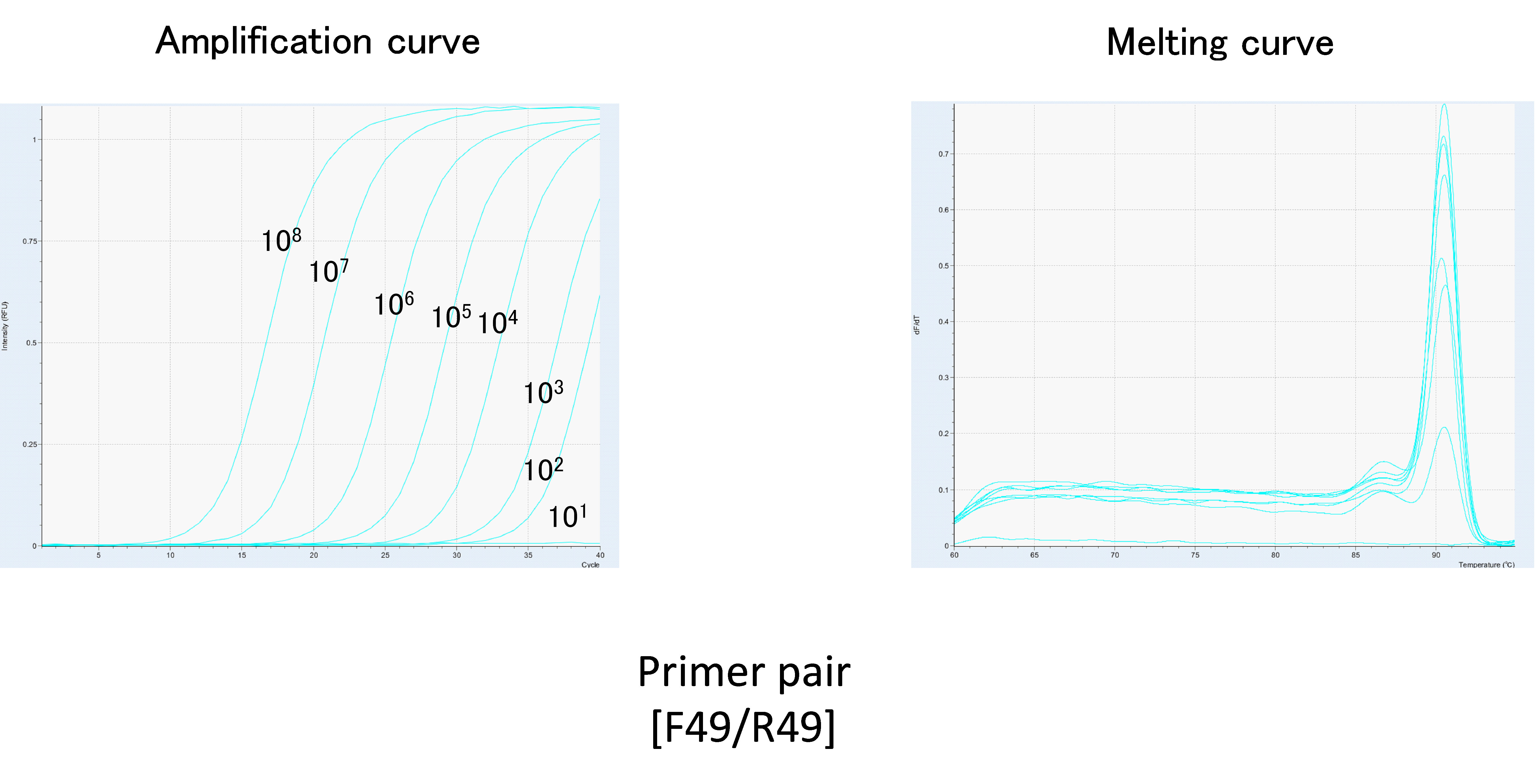

Supplement: Supplementary file 1 — Figure S1. Quantitative real‐time PCR (qPCR) in an acute toxicity test in beagle dogs with single injections of EHMK‐51‐35 carrier cells infected with AdE3‐midkine and Ad‐cGM‐CSF. (A) Specific primers for AdE3‐midkine, AdE3, Ad‐cGM‐CSF and Ad‐fGM‐CSF in qPCR. Amplification and melting curves of qPCR. (B) 10 to 108 copies of AdE3‐midkine with AdE3‐midkine‐specific primers. (C) 108 copies of AdE3‐midkine, AdE3, Ad‐cGM‐CSF and Ad‐fGM‐CSF with AdE3‐midkine‐specific primers. (D) 108 copies of AdE3‐midkine, AdE3, Ad‐cGM‐CSF and Ad‐fGM‐CSF with AE3‐specific primers. (E) 108 copies of AdE3‐midkine, AdE3, Ad‐cGM‐CSF and Ad‐fGM‐CSF with Ad‐cGM‐CSF‐specific primers. (F) 108 copies of AdE3‐midkine, AdE3, Ad‐cGM‐CSF and Ad‐fGM‐CSF with Ad‐fGM‐CSF‐specific primers. Figure S2. Blood analysis results in an acute toxicity test in beagle dogs with single injections of AdE3‐midkine. (A, B) Complete blood count. (C–E) blood chemistry. (F) hemostatic function test results. Figure S3. (A, B) Blood chemistry analysis in chronic toxicity test of EHMK‐51‐35 carrier cells infected with AdE3‐midkine in rabbits with VX2 tumors. Five intratumoral injections were performed. [file JGM-21-na-s001.zip › JGM3064-sup-0002-Fig S1B.tif]

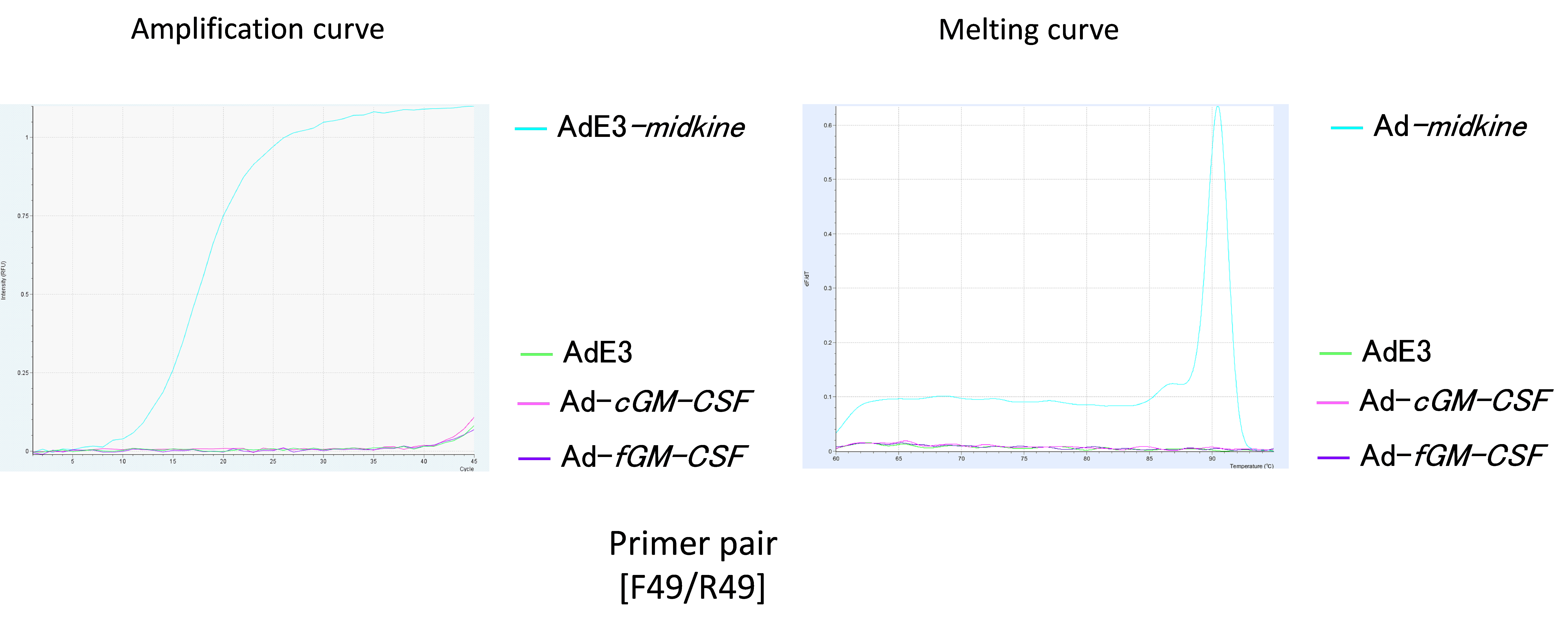

Supplement: Supplementary file 1 — Figure S1. Quantitative real‐time PCR (qPCR) in an acute toxicity test in beagle dogs with single injections of EHMK‐51‐35 carrier cells infected with AdE3‐midkine and Ad‐cGM‐CSF. (A) Specific primers for AdE3‐midkine, AdE3, Ad‐cGM‐CSF and Ad‐fGM‐CSF in qPCR. Amplification and melting curves of qPCR. (B) 10 to 108 copies of AdE3‐midkine with AdE3‐midkine‐specific primers. (C) 108 copies of AdE3‐midkine, AdE3, Ad‐cGM‐CSF and Ad‐fGM‐CSF with AdE3‐midkine‐specific primers. (D) 108 copies of AdE3‐midkine, AdE3, Ad‐cGM‐CSF and Ad‐fGM‐CSF with AE3‐specific primers. (E) 108 copies of AdE3‐midkine, AdE3, Ad‐cGM‐CSF and Ad‐fGM‐CSF with Ad‐cGM‐CSF‐specific primers. (F) 108 copies of AdE3‐midkine, AdE3, Ad‐cGM‐CSF and Ad‐fGM‐CSF with Ad‐fGM‐CSF‐specific primers. Figure S2. Blood analysis results in an acute toxicity test in beagle dogs with single injections of AdE3‐midkine. (A, B) Complete blood count. (C–E) blood chemistry. (F) hemostatic function test results. Figure S3. (A, B) Blood chemistry analysis in chronic toxicity test of EHMK‐51‐35 carrier cells infected with AdE3‐midkine in rabbits with VX2 tumors. Five intratumoral injections were performed. [file JGM-21-na-s001.zip › JGM3064-sup-0003-FIg S1C.tif]

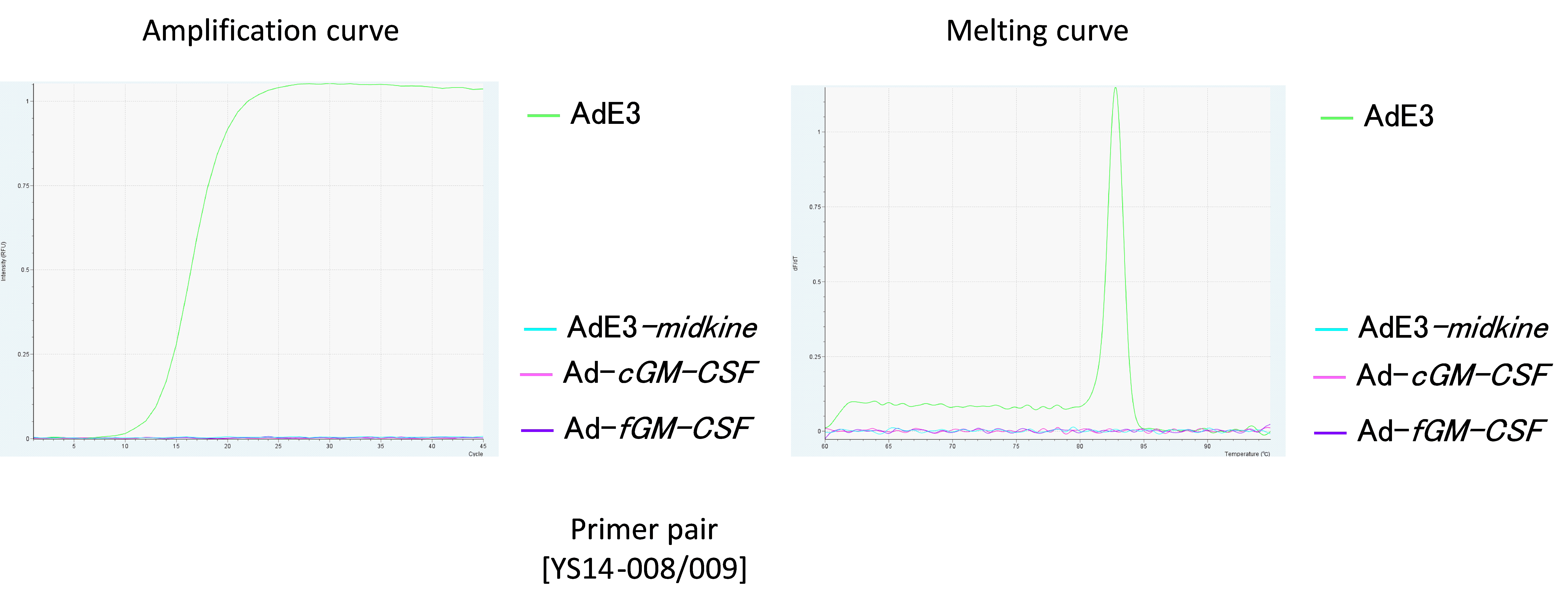

Supplement: Supplementary file 1 — Figure S1. Quantitative real‐time PCR (qPCR) in an acute toxicity test in beagle dogs with single injections of EHMK‐51‐35 carrier cells infected with AdE3‐midkine and Ad‐cGM‐CSF. (A) Specific primers for AdE3‐midkine, AdE3, Ad‐cGM‐CSF and Ad‐fGM‐CSF in qPCR. Amplification and melting curves of qPCR. (B) 10 to 108 copies of AdE3‐midkine with AdE3‐midkine‐specific primers. (C) 108 copies of AdE3‐midkine, AdE3, Ad‐cGM‐CSF and Ad‐fGM‐CSF with AdE3‐midkine‐specific primers. (D) 108 copies of AdE3‐midkine, AdE3, Ad‐cGM‐CSF and Ad‐fGM‐CSF with AE3‐specific primers. (E) 108 copies of AdE3‐midkine, AdE3, Ad‐cGM‐CSF and Ad‐fGM‐CSF with Ad‐cGM‐CSF‐specific primers. (F) 108 copies of AdE3‐midkine, AdE3, Ad‐cGM‐CSF and Ad‐fGM‐CSF with Ad‐fGM‐CSF‐specific primers. Figure S2. Blood analysis results in an acute toxicity test in beagle dogs with single injections of AdE3‐midkine. (A, B) Complete blood count. (C–E) blood chemistry. (F) hemostatic function test results. Figure S3. (A, B) Blood chemistry analysis in chronic toxicity test of EHMK‐51‐35 carrier cells infected with AdE3‐midkine in rabbits with VX2 tumors. Five intratumoral injections were performed. [file JGM-21-na-s001.zip › JGM3064-sup-0004-Fig S1D.tif]

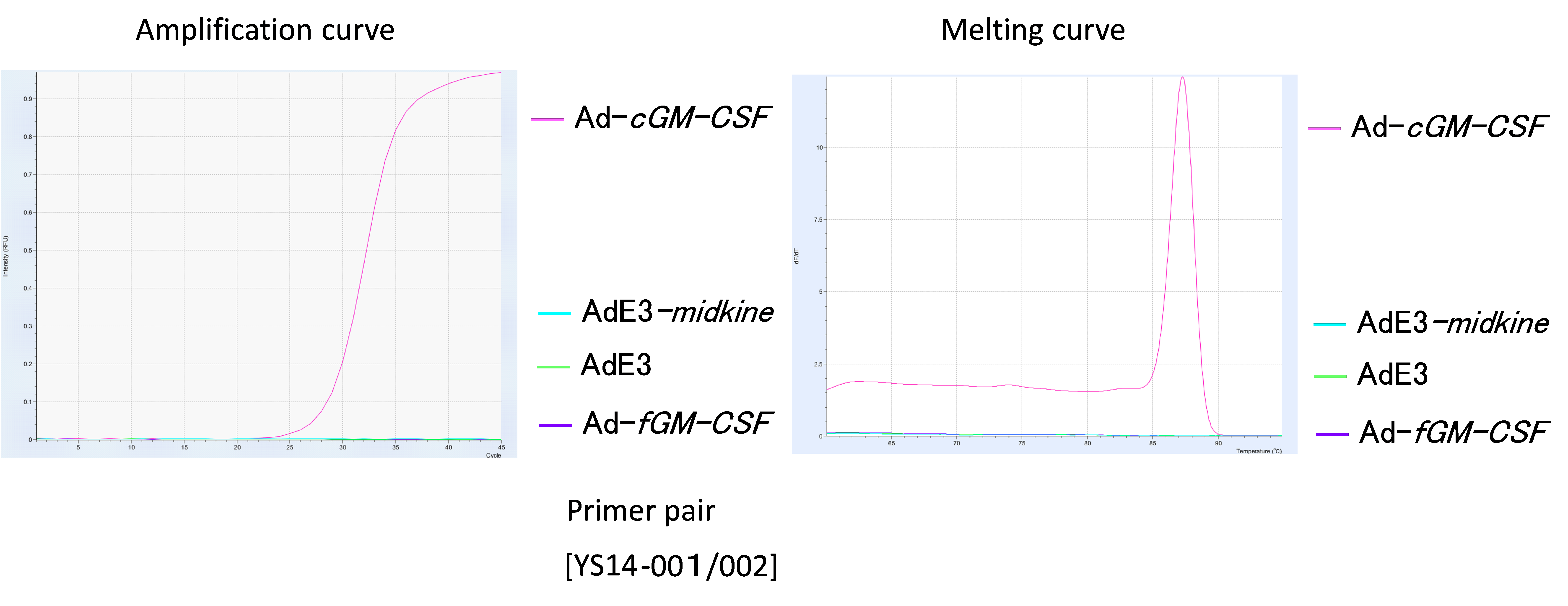

Supplement: Supplementary file 1 — Figure S1. Quantitative real‐time PCR (qPCR) in an acute toxicity test in beagle dogs with single injections of EHMK‐51‐35 carrier cells infected with AdE3‐midkine and Ad‐cGM‐CSF. (A) Specific primers for AdE3‐midkine, AdE3, Ad‐cGM‐CSF and Ad‐fGM‐CSF in qPCR. Amplification and melting curves of qPCR. (B) 10 to 108 copies of AdE3‐midkine with AdE3‐midkine‐specific primers. (C) 108 copies of AdE3‐midkine, AdE3, Ad‐cGM‐CSF and Ad‐fGM‐CSF with AdE3‐midkine‐specific primers. (D) 108 copies of AdE3‐midkine, AdE3, Ad‐cGM‐CSF and Ad‐fGM‐CSF with AE3‐specific primers. (E) 108 copies of AdE3‐midkine, AdE3, Ad‐cGM‐CSF and Ad‐fGM‐CSF with Ad‐cGM‐CSF‐specific primers. (F) 108 copies of AdE3‐midkine, AdE3, Ad‐cGM‐CSF and Ad‐fGM‐CSF with Ad‐fGM‐CSF‐specific primers. Figure S2. Blood analysis results in an acute toxicity test in beagle dogs with single injections of AdE3‐midkine. (A, B) Complete blood count. (C–E) blood chemistry. (F) hemostatic function test results. Figure S3. (A, B) Blood chemistry analysis in chronic toxicity test of EHMK‐51‐35 carrier cells infected with AdE3‐midkine in rabbits with VX2 tumors. Five intratumoral injections were performed. [file JGM-21-na-s001.zip › JGM3064-sup-0005-Fig S1E.tif]

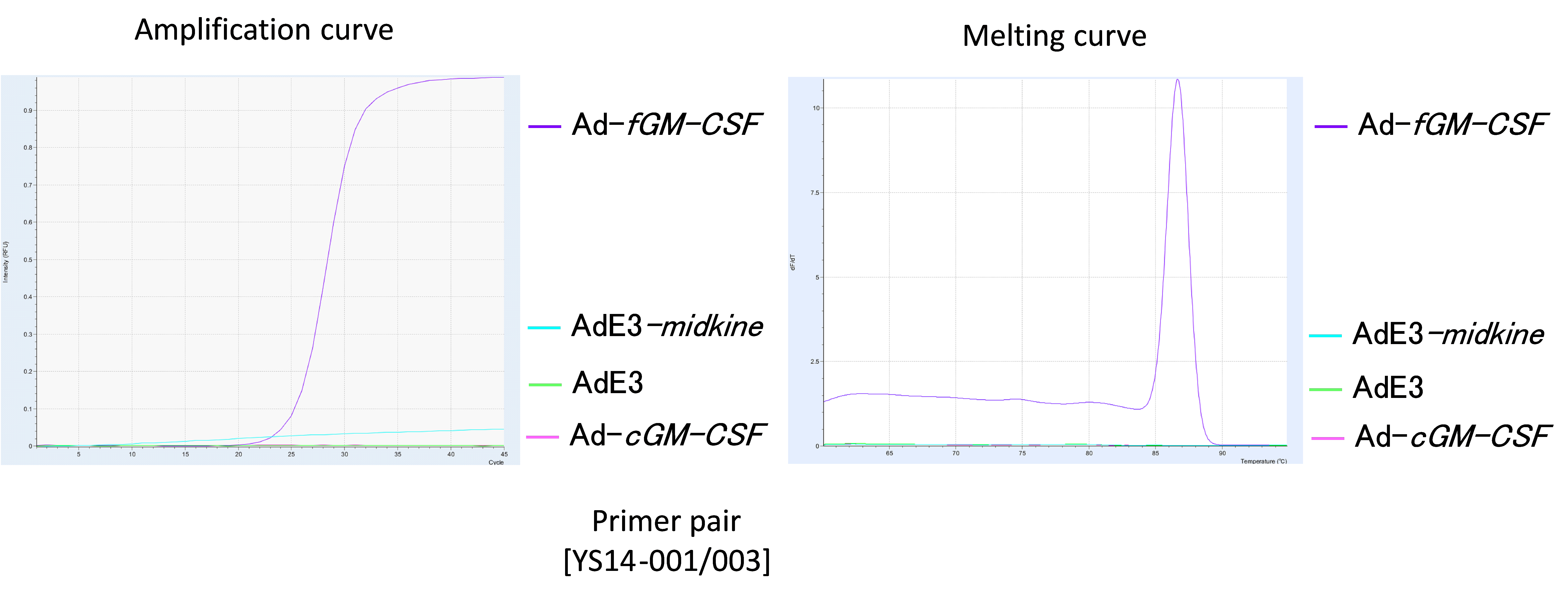

Supplement: Supplementary file 1 — Figure S1. Quantitative real‐time PCR (qPCR) in an acute toxicity test in beagle dogs with single injections of EHMK‐51‐35 carrier cells infected with AdE3‐midkine and Ad‐cGM‐CSF. (A) Specific primers for AdE3‐midkine, AdE3, Ad‐cGM‐CSF and Ad‐fGM‐CSF in qPCR. Amplification and melting curves of qPCR. (B) 10 to 108 copies of AdE3‐midkine with AdE3‐midkine‐specific primers. (C) 108 copies of AdE3‐midkine, AdE3, Ad‐cGM‐CSF and Ad‐fGM‐CSF with AdE3‐midkine‐specific primers. (D) 108 copies of AdE3‐midkine, AdE3, Ad‐cGM‐CSF and Ad‐fGM‐CSF with AE3‐specific primers. (E) 108 copies of AdE3‐midkine, AdE3, Ad‐cGM‐CSF and Ad‐fGM‐CSF with Ad‐cGM‐CSF‐specific primers. (F) 108 copies of AdE3‐midkine, AdE3, Ad‐cGM‐CSF and Ad‐fGM‐CSF with Ad‐fGM‐CSF‐specific primers. Figure S2. Blood analysis results in an acute toxicity test in beagle dogs with single injections of AdE3‐midkine. (A, B) Complete blood count. (C–E) blood chemistry. (F) hemostatic function test results. Figure S3. (A, B) Blood chemistry analysis in chronic toxicity test of EHMK‐51‐35 carrier cells infected with AdE3‐midkine in rabbits with VX2 tumors. Five intratumoral injections were performed. [file JGM-21-na-s001.zip › JGM3064-sup-0006-Fig S1F.tif]

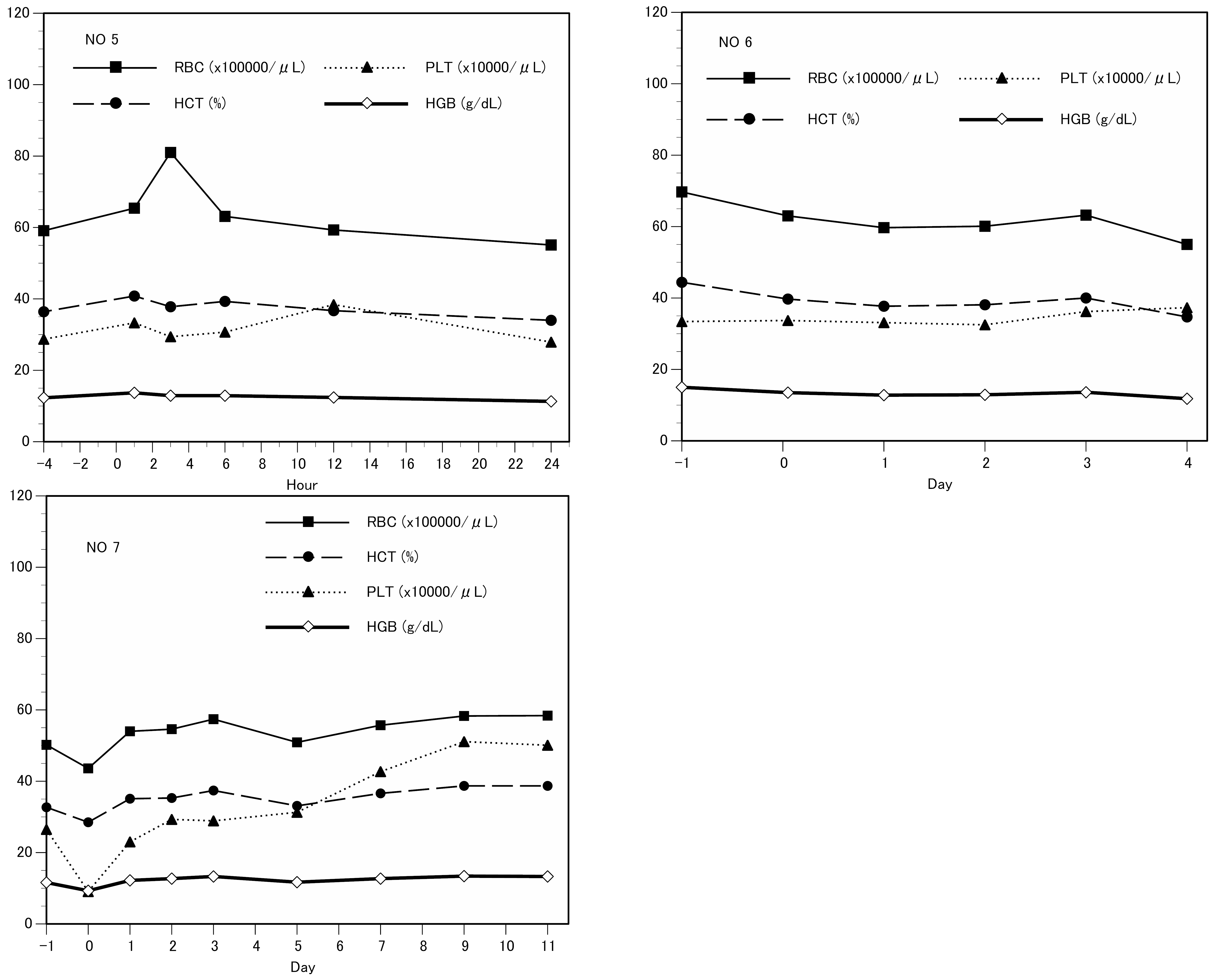

Supplement: Supplementary file 1 — Figure S1. Quantitative real‐time PCR (qPCR) in an acute toxicity test in beagle dogs with single injections of EHMK‐51‐35 carrier cells infected with AdE3‐midkine and Ad‐cGM‐CSF. (A) Specific primers for AdE3‐midkine, AdE3, Ad‐cGM‐CSF and Ad‐fGM‐CSF in qPCR. Amplification and melting curves of qPCR. (B) 10 to 108 copies of AdE3‐midkine with AdE3‐midkine‐specific primers. (C) 108 copies of AdE3‐midkine, AdE3, Ad‐cGM‐CSF and Ad‐fGM‐CSF with AdE3‐midkine‐specific primers. (D) 108 copies of AdE3‐midkine, AdE3, Ad‐cGM‐CSF and Ad‐fGM‐CSF with AE3‐specific primers. (E) 108 copies of AdE3‐midkine, AdE3, Ad‐cGM‐CSF and Ad‐fGM‐CSF with Ad‐cGM‐CSF‐specific primers. (F) 108 copies of AdE3‐midkine, AdE3, Ad‐cGM‐CSF and Ad‐fGM‐CSF with Ad‐fGM‐CSF‐specific primers. Figure S2. Blood analysis results in an acute toxicity test in beagle dogs with single injections of AdE3‐midkine. (A, B) Complete blood count. (C–E) blood chemistry. (F) hemostatic function test results. Figure S3. (A, B) Blood chemistry analysis in chronic toxicity test of EHMK‐51‐35 carrier cells infected with AdE3‐midkine in rabbits with VX2 tumors. Five intratumoral injections were performed. [file JGM-21-na-s001.zip › JGM3064-sup-0007-Fig S2A.tif]

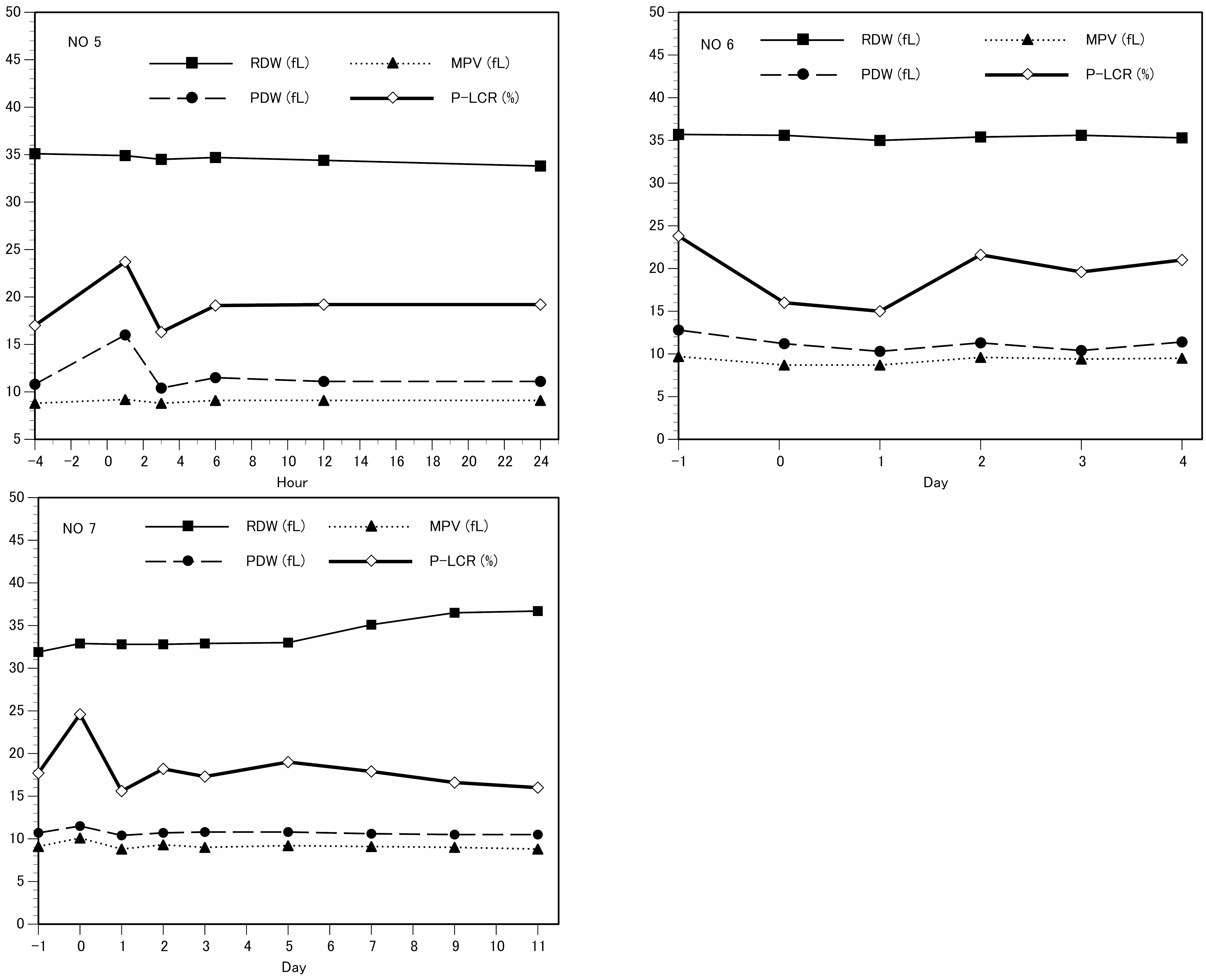

Supplement: Supplementary file 1 — Figure S1. Quantitative real‐time PCR (qPCR) in an acute toxicity test in beagle dogs with single injections of EHMK‐51‐35 carrier cells infected with AdE3‐midkine and Ad‐cGM‐CSF. (A) Specific primers for AdE3‐midkine, AdE3, Ad‐cGM‐CSF and Ad‐fGM‐CSF in qPCR. Amplification and melting curves of qPCR. (B) 10 to 108 copies of AdE3‐midkine with AdE3‐midkine‐specific primers. (C) 108 copies of AdE3‐midkine, AdE3, Ad‐cGM‐CSF and Ad‐fGM‐CSF with AdE3‐midkine‐specific primers. (D) 108 copies of AdE3‐midkine, AdE3, Ad‐cGM‐CSF and Ad‐fGM‐CSF with AE3‐specific primers. (E) 108 copies of AdE3‐midkine, AdE3, Ad‐cGM‐CSF and Ad‐fGM‐CSF with Ad‐cGM‐CSF‐specific primers. (F) 108 copies of AdE3‐midkine, AdE3, Ad‐cGM‐CSF and Ad‐fGM‐CSF with Ad‐fGM‐CSF‐specific primers. Figure S2. Blood analysis results in an acute toxicity test in beagle dogs with single injections of AdE3‐midkine. (A, B) Complete blood count. (C–E) blood chemistry. (F) hemostatic function test results. Figure S3. (A, B) Blood chemistry analysis in chronic toxicity test of EHMK‐51‐35 carrier cells infected with AdE3‐midkine in rabbits with VX2 tumors. Five intratumoral injections were performed. [file JGM-21-na-s001.zip › JGM3064-sup-0008-Fig S2B.tif]

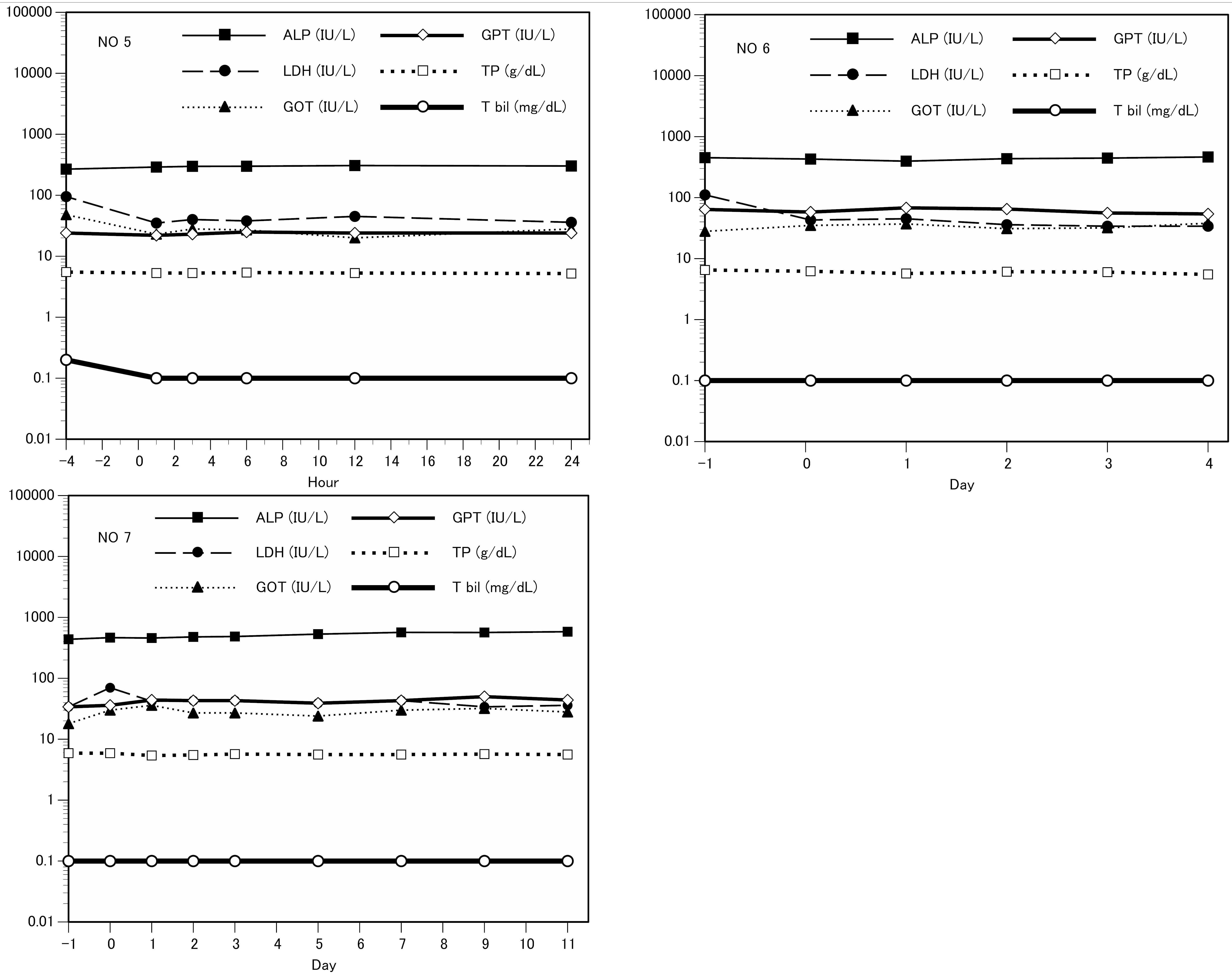

Supplement: Supplementary file 1 — Figure S1. Quantitative real‐time PCR (qPCR) in an acute toxicity test in beagle dogs with single injections of EHMK‐51‐35 carrier cells infected with AdE3‐midkine and Ad‐cGM‐CSF. (A) Specific primers for AdE3‐midkine, AdE3, Ad‐cGM‐CSF and Ad‐fGM‐CSF in qPCR. Amplification and melting curves of qPCR. (B) 10 to 108 copies of AdE3‐midkine with AdE3‐midkine‐specific primers. (C) 108 copies of AdE3‐midkine, AdE3, Ad‐cGM‐CSF and Ad‐fGM‐CSF with AdE3‐midkine‐specific primers. (D) 108 copies of AdE3‐midkine, AdE3, Ad‐cGM‐CSF and Ad‐fGM‐CSF with AE3‐specific primers. (E) 108 copies of AdE3‐midkine, AdE3, Ad‐cGM‐CSF and Ad‐fGM‐CSF with Ad‐cGM‐CSF‐specific primers. (F) 108 copies of AdE3‐midkine, AdE3, Ad‐cGM‐CSF and Ad‐fGM‐CSF with Ad‐fGM‐CSF‐specific primers. Figure S2. Blood analysis results in an acute toxicity test in beagle dogs with single injections of AdE3‐midkine. (A, B) Complete blood count. (C–E) blood chemistry. (F) hemostatic function test results. Figure S3. (A, B) Blood chemistry analysis in chronic toxicity test of EHMK‐51‐35 carrier cells infected with AdE3‐midkine in rabbits with VX2 tumors. Five intratumoral injections were performed. [file JGM-21-na-s001.zip › JGM3064-sup-0009-Fig S2C.tif]

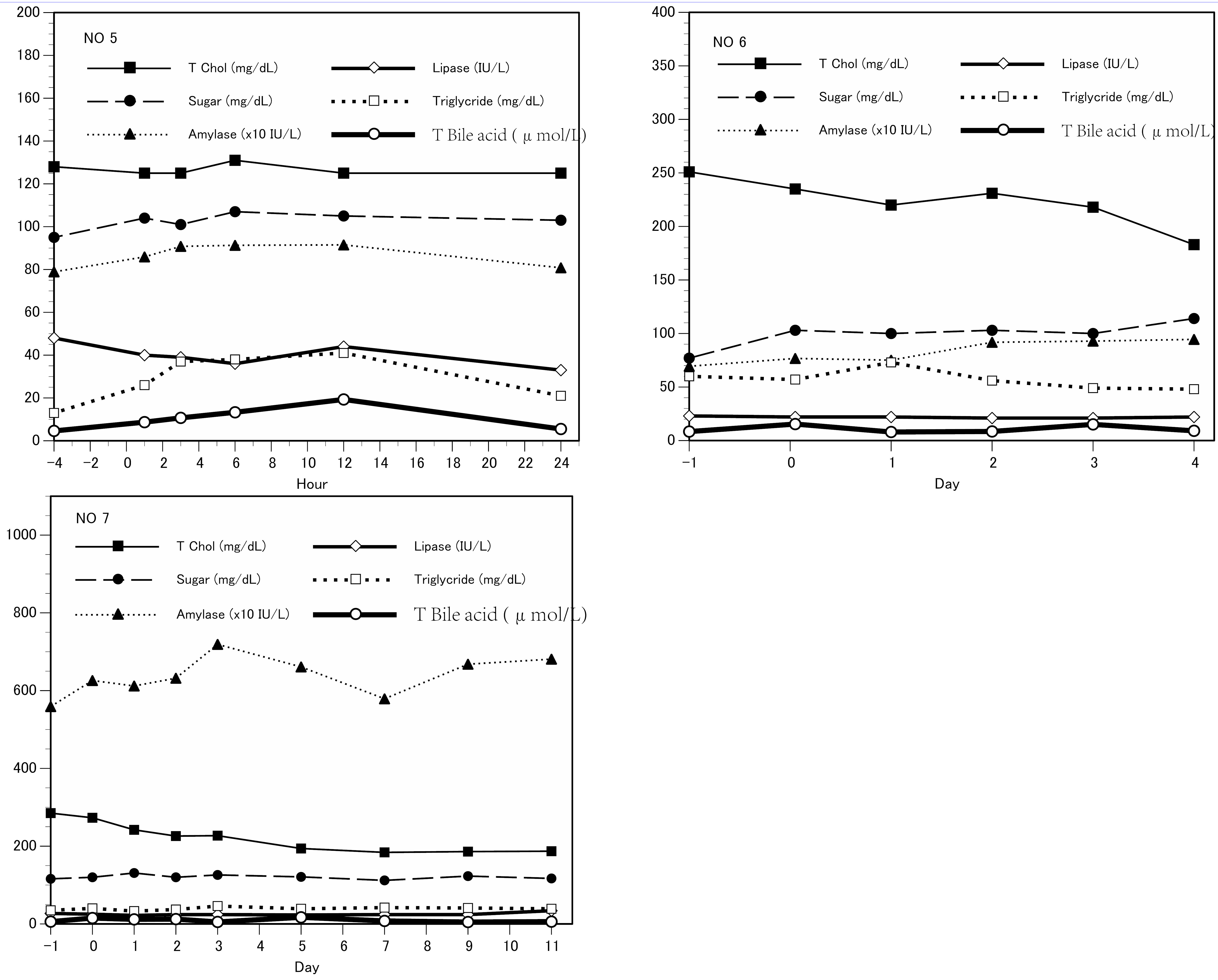

Supplement: Supplementary file 1 — Figure S1. Quantitative real‐time PCR (qPCR) in an acute toxicity test in beagle dogs with single injections of EHMK‐51‐35 carrier cells infected with AdE3‐midkine and Ad‐cGM‐CSF. (A) Specific primers for AdE3‐midkine, AdE3, Ad‐cGM‐CSF and Ad‐fGM‐CSF in qPCR. Amplification and melting curves of qPCR. (B) 10 to 108 copies of AdE3‐midkine with AdE3‐midkine‐specific primers. (C) 108 copies of AdE3‐midkine, AdE3, Ad‐cGM‐CSF and Ad‐fGM‐CSF with AdE3‐midkine‐specific primers. (D) 108 copies of AdE3‐midkine, AdE3, Ad‐cGM‐CSF and Ad‐fGM‐CSF with AE3‐specific primers. (E) 108 copies of AdE3‐midkine, AdE3, Ad‐cGM‐CSF and Ad‐fGM‐CSF with Ad‐cGM‐CSF‐specific primers. (F) 108 copies of AdE3‐midkine, AdE3, Ad‐cGM‐CSF and Ad‐fGM‐CSF with Ad‐fGM‐CSF‐specific primers. Figure S2. Blood analysis results in an acute toxicity test in beagle dogs with single injections of AdE3‐midkine. (A, B) Complete blood count. (C–E) blood chemistry. (F) hemostatic function test results. Figure S3. (A, B) Blood chemistry analysis in chronic toxicity test of EHMK‐51‐35 carrier cells infected with AdE3‐midkine in rabbits with VX2 tumors. Five intratumoral injections were performed. [file JGM-21-na-s001.zip › JGM3064-sup-0010-Fig S2D.tif]

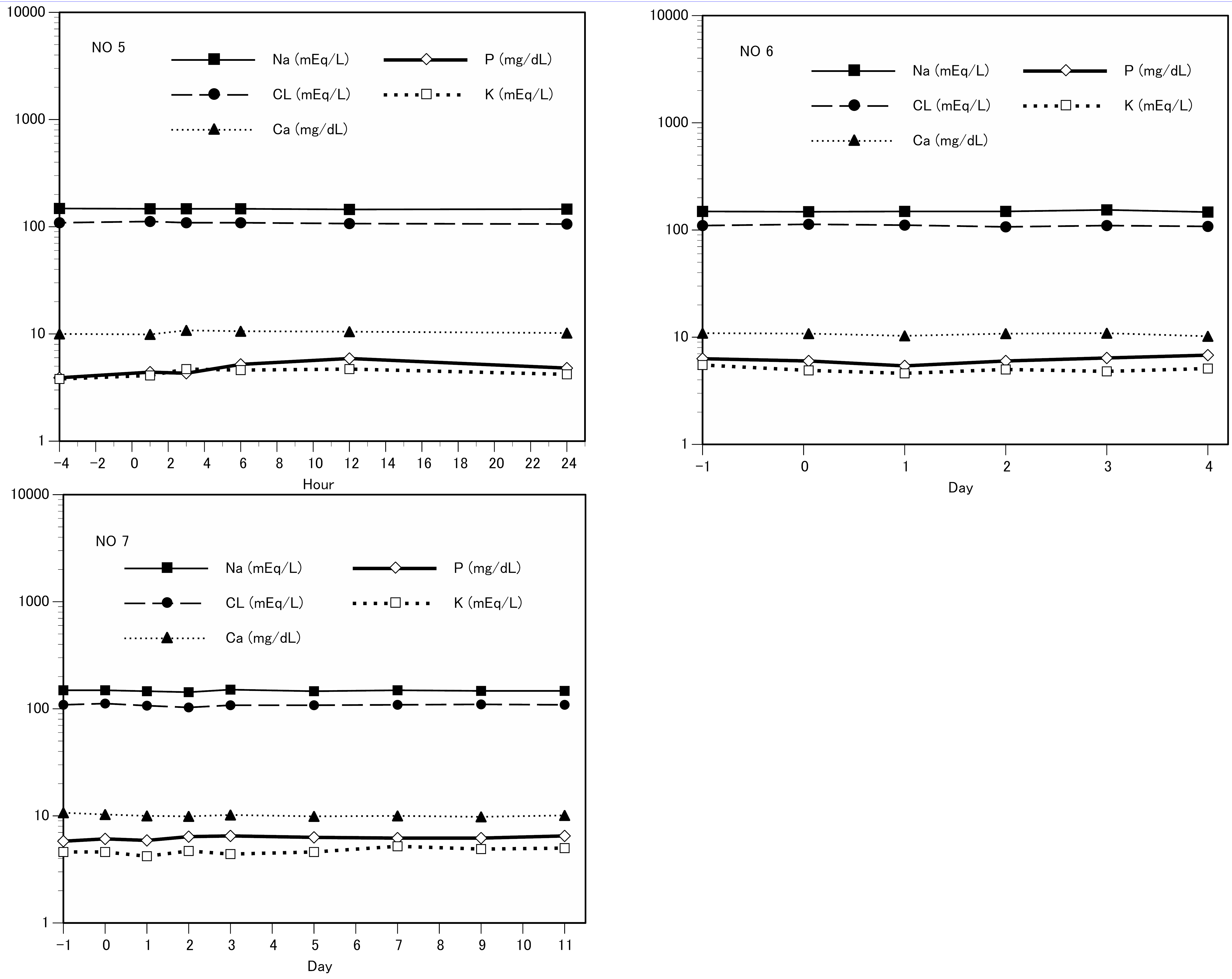

Supplement: Supplementary file 1 — Figure S1. Quantitative real‐time PCR (qPCR) in an acute toxicity test in beagle dogs with single injections of EHMK‐51‐35 carrier cells infected with AdE3‐midkine and Ad‐cGM‐CSF. (A) Specific primers for AdE3‐midkine, AdE3, Ad‐cGM‐CSF and Ad‐fGM‐CSF in qPCR. Amplification and melting curves of qPCR. (B) 10 to 108 copies of AdE3‐midkine with AdE3‐midkine‐specific primers. (C) 108 copies of AdE3‐midkine, AdE3, Ad‐cGM‐CSF and Ad‐fGM‐CSF with AdE3‐midkine‐specific primers. (D) 108 copies of AdE3‐midkine, AdE3, Ad‐cGM‐CSF and Ad‐fGM‐CSF with AE3‐specific primers. (E) 108 copies of AdE3‐midkine, AdE3, Ad‐cGM‐CSF and Ad‐fGM‐CSF with Ad‐cGM‐CSF‐specific primers. (F) 108 copies of AdE3‐midkine, AdE3, Ad‐cGM‐CSF and Ad‐fGM‐CSF with Ad‐fGM‐CSF‐specific primers. Figure S2. Blood analysis results in an acute toxicity test in beagle dogs with single injections of AdE3‐midkine. (A, B) Complete blood count. (C–E) blood chemistry. (F) hemostatic function test results. Figure S3. (A, B) Blood chemistry analysis in chronic toxicity test of EHMK‐51‐35 carrier cells infected with AdE3‐midkine in rabbits with VX2 tumors. Five intratumoral injections were performed. [file JGM-21-na-s001.zip › JGM3064-sup-0011-Fig S2E.tif]

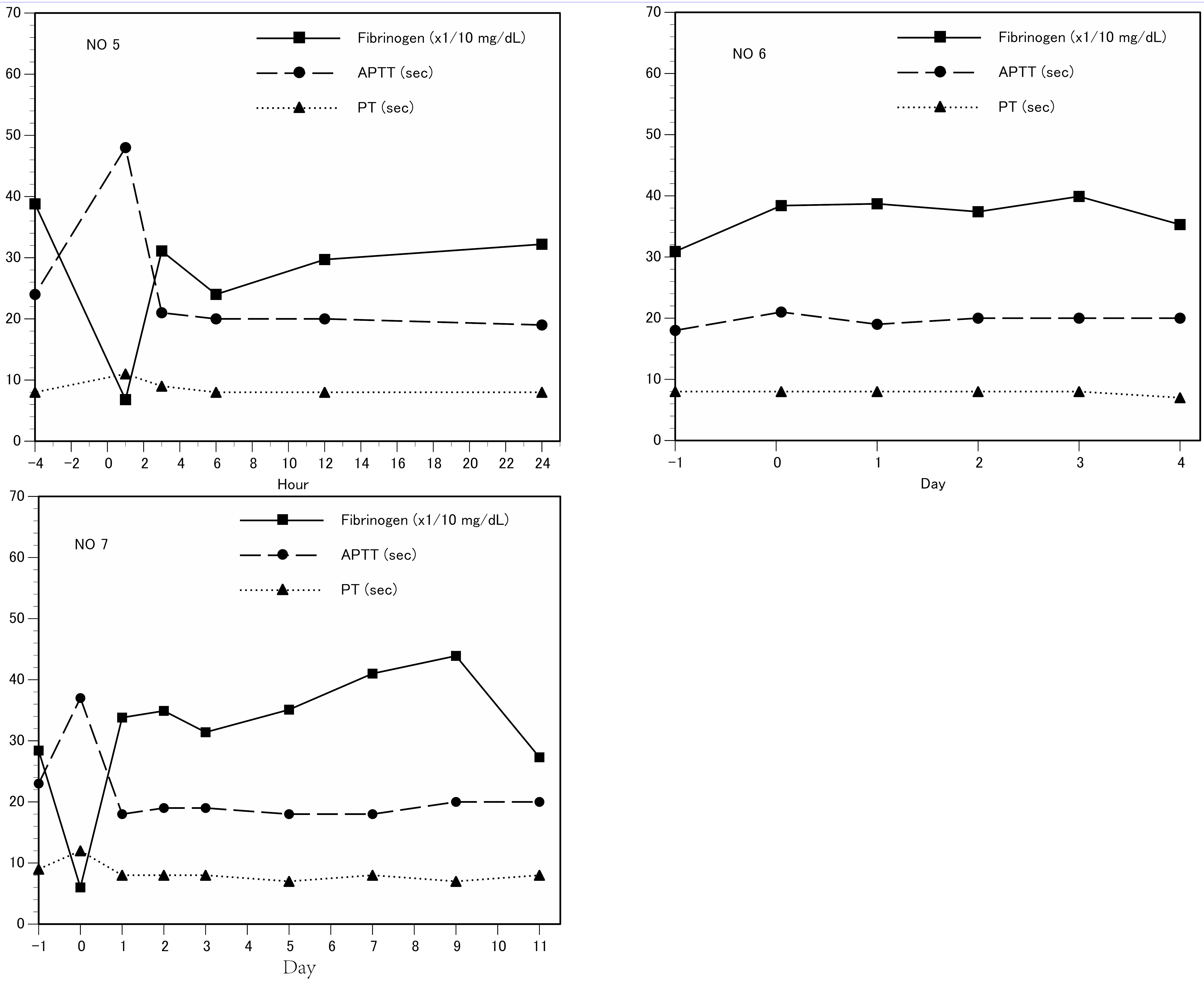

Supplement: Supplementary file 1 — Figure S1. Quantitative real‐time PCR (qPCR) in an acute toxicity test in beagle dogs with single injections of EHMK‐51‐35 carrier cells infected with AdE3‐midkine and Ad‐cGM‐CSF. (A) Specific primers for AdE3‐midkine, AdE3, Ad‐cGM‐CSF and Ad‐fGM‐CSF in qPCR. Amplification and melting curves of qPCR. (B) 10 to 108 copies of AdE3‐midkine with AdE3‐midkine‐specific primers. (C) 108 copies of AdE3‐midkine, AdE3, Ad‐cGM‐CSF and Ad‐fGM‐CSF with AdE3‐midkine‐specific primers. (D) 108 copies of AdE3‐midkine, AdE3, Ad‐cGM‐CSF and Ad‐fGM‐CSF with AE3‐specific primers. (E) 108 copies of AdE3‐midkine, AdE3, Ad‐cGM‐CSF and Ad‐fGM‐CSF with Ad‐cGM‐CSF‐specific primers. (F) 108 copies of AdE3‐midkine, AdE3, Ad‐cGM‐CSF and Ad‐fGM‐CSF with Ad‐fGM‐CSF‐specific primers. Figure S2. Blood analysis results in an acute toxicity test in beagle dogs with single injections of AdE3‐midkine. (A, B) Complete blood count. (C–E) blood chemistry. (F) hemostatic function test results. Figure S3. (A, B) Blood chemistry analysis in chronic toxicity test of EHMK‐51‐35 carrier cells infected with AdE3‐midkine in rabbits with VX2 tumors. Five intratumoral injections were performed. [file JGM-21-na-s001.zip › JGM3064-sup-0012-Fig S2F-.tif]

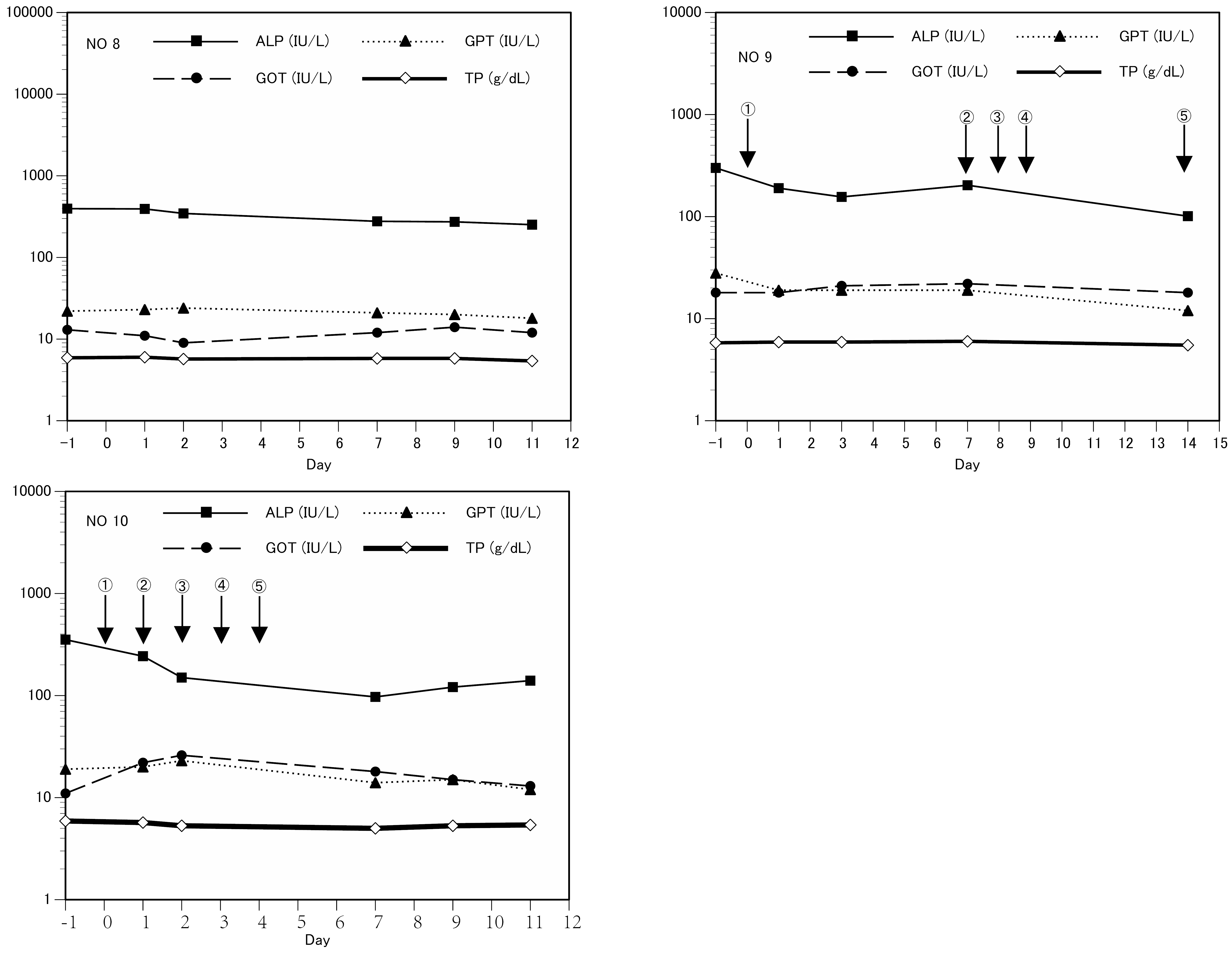

Supplement: Supplementary file 1 — Figure S1. Quantitative real‐time PCR (qPCR) in an acute toxicity test in beagle dogs with single injections of EHMK‐51‐35 carrier cells infected with AdE3‐midkine and Ad‐cGM‐CSF. (A) Specific primers for AdE3‐midkine, AdE3, Ad‐cGM‐CSF and Ad‐fGM‐CSF in qPCR. Amplification and melting curves of qPCR. (B) 10 to 108 copies of AdE3‐midkine with AdE3‐midkine‐specific primers. (C) 108 copies of AdE3‐midkine, AdE3, Ad‐cGM‐CSF and Ad‐fGM‐CSF with AdE3‐midkine‐specific primers. (D) 108 copies of AdE3‐midkine, AdE3, Ad‐cGM‐CSF and Ad‐fGM‐CSF with AE3‐specific primers. (E) 108 copies of AdE3‐midkine, AdE3, Ad‐cGM‐CSF and Ad‐fGM‐CSF with Ad‐cGM‐CSF‐specific primers. (F) 108 copies of AdE3‐midkine, AdE3, Ad‐cGM‐CSF and Ad‐fGM‐CSF with Ad‐fGM‐CSF‐specific primers. Figure S2. Blood analysis results in an acute toxicity test in beagle dogs with single injections of AdE3‐midkine. (A, B) Complete blood count. (C–E) blood chemistry. (F) hemostatic function test results. Figure S3. (A, B) Blood chemistry analysis in chronic toxicity test of EHMK‐51‐35 carrier cells infected with AdE3‐midkine in rabbits with VX2 tumors. Five intratumoral injections were performed. [file JGM-21-na-s001.zip › JGM3064-sup-0013-Fig S3A.tif]

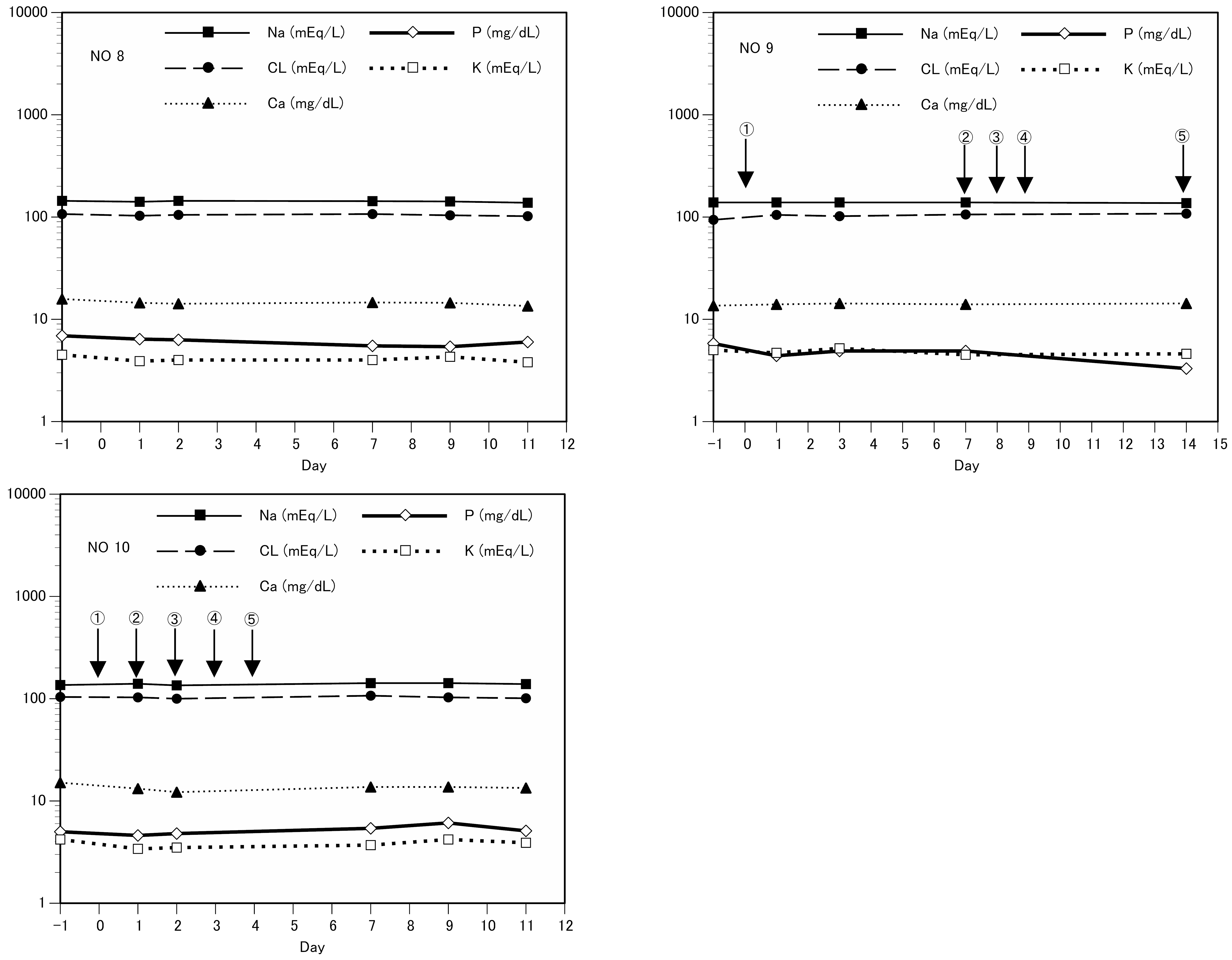

Supplement: Supplementary file 1 — Figure S1. Quantitative real‐time PCR (qPCR) in an acute toxicity test in beagle dogs with single injections of EHMK‐51‐35 carrier cells infected with AdE3‐midkine and Ad‐cGM‐CSF. (A) Specific primers for AdE3‐midkine, AdE3, Ad‐cGM‐CSF and Ad‐fGM‐CSF in qPCR. Amplification and melting curves of qPCR. (B) 10 to 108 copies of AdE3‐midkine with AdE3‐midkine‐specific primers. (C) 108 copies of AdE3‐midkine, AdE3, Ad‐cGM‐CSF and Ad‐fGM‐CSF with AdE3‐midkine‐specific primers. (D) 108 copies of AdE3‐midkine, AdE3, Ad‐cGM‐CSF and Ad‐fGM‐CSF with AE3‐specific primers. (E) 108 copies of AdE3‐midkine, AdE3, Ad‐cGM‐CSF and Ad‐fGM‐CSF with Ad‐cGM‐CSF‐specific primers. (F) 108 copies of AdE3‐midkine, AdE3, Ad‐cGM‐CSF and Ad‐fGM‐CSF with Ad‐fGM‐CSF‐specific primers. Figure S2. Blood analysis results in an acute toxicity test in beagle dogs with single injections of AdE3‐midkine. (A, B) Complete blood count. (C–E) blood chemistry. (F) hemostatic function test results. Figure S3. (A, B) Blood chemistry analysis in chronic toxicity test of EHMK‐51‐35 carrier cells infected with AdE3‐midkine in rabbits with VX2 tumors. Five intratumoral injections were performed. [file JGM-21-na-s001.zip › JGM3064-sup-0014-Fig S3B.tif]
